# The Emerging Role of Long Non-Coding RNAs and MicroRNAs in Neurodegenerative Diseases: A Perspective of Machine Learning

**DOI:** 10.3390/biom11081132

**Published:** 2021-07-31

**Authors:** Ángela García-Fonseca, Cynthia Martin-Jimenez, George E. Barreto, Andres Felipe Aristizábal Pachón, Janneth González

**Affiliations:** 1 Departamento de Nutrición y Bioquímica, Facultad de Ciencias, Pontificia Universidad Javeriana, Bogotá 110231, Colombia; aygarciafo@unal.edu.co (Á.G.-F.); martin.c@javeriana.edu.co (C.M.-J.); andres_aristizabal@javeriana.edu.co (A.F.A.P.); 2 Department of Biological Sciences, University of Limerick, V94 T9PX Limerick, Ireland; George.Barreto@ul.ie

**Keywords:** miRNA, long non-coding RNA, biomarker, neurodegenerative disease, artificial intelligence, machine learning

## Abstract

Neurodegenerative diseases (NDs) are characterized by progressive neuronal dysfunction and death of brain cells population. As the early manifestations of NDs are similar, their symptoms are difficult to distinguish, making the timely detection and discrimination of each neurodegenerative disorder a priority. Several investigations have revealed the importance of microRNAs and long non-coding RNAs in neurodevelopment, brain function, maturation, and neuronal activity, as well as its dysregulation involved in many types of neurological diseases. Therefore, the expression pattern of these molecules in the different NDs have gained significant attention to improve the diagnostic and treatment at earlier stages. In this sense, we gather the different microRNAs and long non-coding RNAs that have been reported as dysregulated in each disorder. Since there are a vast number of non-coding RNAs altered in NDs, some sort of synthesis, filtering and organization method should be applied to extract the most relevant information. Hence, machine learning is considered as an important tool for this purpose since it can classify expression profiles of non-coding RNAs between healthy and sick people. Therefore, we deepen in this branch of computer science, its different methods, and its meaningful application in the diagnosis of NDs from the dysregulated non-coding RNAs. In addition, we demonstrate the relevance of machine learning in NDs from the description of different investigations that showed an accuracy between 85% to 95% in the detection of the disease with this tool. All of these denote that artificial intelligence could be an excellent alternative to help the clinical diagnosis and facilitate the identification diseases in early stages based on non-coding RNAs.

## 1. Introduction

The neurodegenerative diseases (NDs) are characterized by progressive and irreversible loss of neurons and other brain cells, resulting in a set of functional alterations in the central nervous system (CNS) [1]. The most common risk factors for the development of NDs are age, inflammation, obesity and genetic alterations [2,3]. The specificity of NDs’ diagnosis is very low due to some similarities in their early symptoms, along with greater clinical variability, making it inefficient and imprecise the implementation of future treatment schemes [4]. It is a well-established fact that dysregulation of non-coding RNAs (nc-RNAs) present only in patients with one type of ND are considered as biomarkers, which could be an alternative to accurately diagnose NDs. Since microRNAs (miRNAs) and long non-coding RNAs (lnc-RNAs) do not code proteins, their importance has been implicated in several biological processes that play a pivotal role in the regulation of cell differentiation, development, proliferation and apoptosis [5,6].

Studies using blood samples and postmortem brain tissue from patients with Parkinson’s and Alzheimer’s disease demonstrated a differential expression of lnc-RNAs as Sox2OT, BC200, BACE1-AS, and NAT-Rad18 or miRNAs such as miR-1, miR-22p, miR-26b-3p and miR-28-3p [7,8,9,10,11]. It has been sought these alterations play a significant role in the control of: ND-related genes expression, changes in the networks or signaling pathways involved in cell physiological functions and the consequent phenotypic manifestation. Since, biomarkers to detect this disorder in preclinical states have not yet been discovered, miRNAs and lnc-RNAs have been considered as possible cellular and druggable targets to predict and treat the neurodegenerative event prior to the manifestation of clinical symptoms and functional impairment [12,13]. Additionally, another advantage of these molecules is their stability and presence in biofluids such as blood, saliva, urine and cerebrospinal fluid [14,15,16], making possible their ease obtaining and identification for an early and more effective diagnosis [17,18,19]. Considering the fact that alterations in miRNAs and lnc-RNAs have been associated to specific NDs, their identification and further study is not an easy task due to technical and experimental limitations. In response to these challenges, scientists have implemented advanced machine learning models for preparing complex assays, performing high-content multi-parametric analysis, and interpreting large, complicated datasets applied to mental health. As such, this recent approach might enable more precise, earlier diagnosis of each NDs on the basis of medical history, and molecular profiles of the patient’s endotype.

Machine learning (ML) is a tool of computer science and artificial intelligence (AI), designed to simulate human intelligence learning from the data and ongoing experience [20]. This technology requires the integration of multiple data sets of biological information, enabling the creation of a statistical model that helps predict some unknown parameter [21]. In the last years, ML has gained a key role in medicine, where it has been important to detect several pathologies. For instance, in cancer or in drug therapy, ML can help to classify tumors or predict personalized drug responses based on the gene expression profile [22,23,24,25]. In case of NDs, the use of ML focuses on finding specific changes in gene expression in the specific disease that allows early diagnosis of the disorder [20]. Hence, a large data set of dysregulated miRNAs and lnc-RNAs in patients with some type of ND can be used as potential biomarkers and employed in machine learning algorithms. In that way, a model can be created to detect each illness, thereby, it is possible to predict the pathology more accurately and differentiate between each ND based on patient’s information.

In this review, we aimed to highlight several unregulated nc-RNAs that have been reported for different types of NDs in various investigations. As there is a very large set of nc-RNAs, this data can provide great information to early diagnosis with ML, making use of algorithms and consequently model development. In this sense, a ND can be detected from the expression profile of nc-RNAs in each patient. To corroborate and understand about this approach, we aimed to describe ML, their types, some methods and their advantages or disadvantages. Moreover, we emphasize this tool with the description of some investigations about the diagnosis of NDs using a ML approach. These studies show a high accuracy in the identification of the disease, which demonstrates that machine learning is a good tool for clinical diagnosis. Finally, we discus sight the importance of AI in health and how it can contribute to improve the diagnosis and treatment of NDs in the near future.

## 2. MicroRNAs and Long Non-Coding RNAs

### 2.1. MicroRNAs

**Figure 1:**
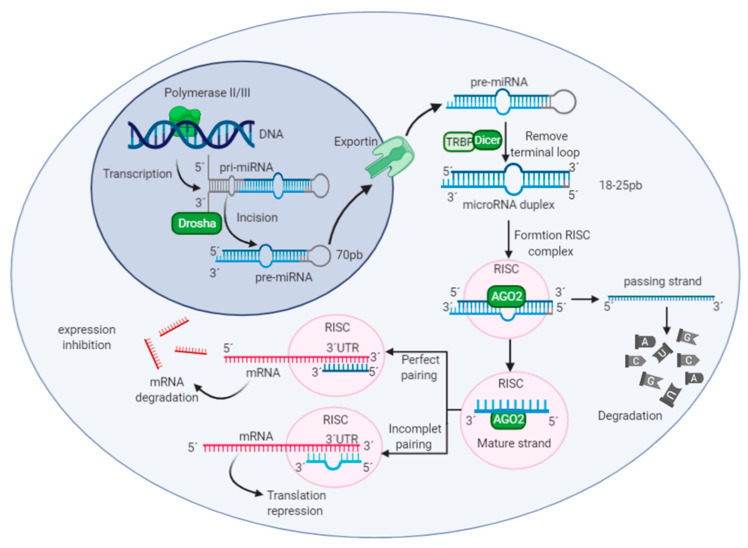
miRNA biogenesis and mechanism of action. The synthesis of these molecules is initiated in the nucleus through the transcription of a DNA region by the enzyme RNA polymerase II/III. The enzyme allows the formation of the primary microRNA (pri-miRNA). The pri-miRNA is a large double strand of RNA with a terminal loop and a single-stranded RNA extension at the other extreme [33,39]. The enzyme Drosha generates an incision in the single-stranded RNA extension of pri-miRNA. This process results in a precursor miRNA (pre-miRNA) of double-strand that has a size of 70 base pairs approximately [40]. Afterward, the pre-miRNA is exported to the cytoplasm by a protein called exportin and the miRNA continues with its maturation procedure. On the cytoplasm, the Dicer enzyme eliminates terminal loop present in the double-stranded pre-miRNA. This produces a microRNA duplex with a size of 18–25 base pairs [41]. Subsequently, the microRNA duplex interacts with the cytoplasmic protein AGO2 which degrades one of the two strands (passing strand) and another strand will be functional strand (mature strand). The mature strand remains bound to the enzyme AGO2, and they are incorporated into the RISC complex. In this complex is generated the union between target mRNA and the mature microRNA [40,42]. The grade of complementarity between miRNA-mRNA determines whether the mRNA is degraded, or the repression of translation is induced [43].

microRNAs are small molecules of RNA not coding for proteins, discovered in the nematode *Caenorhabditis elegans* [26]. In these organisms, the downregulation of LIN-14 protein was involved in the binding of miRNA to the 3′ untranslated region (3′ UTR) of the mRNA target [27]. This binding triggers a negative regulation in the expression of the target gene [28]. Let-7 was the second miRNA found in *C. elegans*, which was involved in processes of larval development [29,30]. In humans, let-7 family expression was detected in tissues like brain, heart, lung, among others and they have been involved in stem cell biology, differentiation and metabolism [31,32]. Therefore, investigations have revealed that miRNAs can regulate the gene expression at post-transcriptional for maintaining cell function [33]. Therefore, investigations have revealed that miRNAs can regulate the gene expression at post-transcriptional for maintaining cell function [33]. Nonetheless, the dysregulation of miRNAs could generate several diseases like cancer, cardiovascular issues and NDs [34,35]. Alterations in miRNAs expression are often given by genomic events in miRNAs sequences, such as point mutations, amplifications, deletion, or transcriptional changes [36]. In addition, enzymes that regulate miRNA biogenesis could also have mutations or downregulations [37,38] (Figure 1).

### 2.2. Long Non-Coding RNAs

Long non-coding RNAs are molecules with size of 200 nucleotides approximately that are not translated to proteins [44]. Lnc-RNAs are originated from intronic, intergenic regions, enhancers, promoters and the opposite strand of protein-coding genes [44]. The H19 and XIST were the first mammalian lnc-RNAs to be identified, yet these lnc-RNAs were not of major interest at the time [45,46]. In 2001, although the complete sequencing of the human genome was published, the investigators discovered that only 1.1% of genome encode proteins and the rest of genome were non-coding RNAs. This highlighted that the functions of these molecules are not entirely clear [47]. Subsequently, the role of lnc-RNAs has been characterized in humans. Studies have shown that these molecules are another way to promote gene regulation and, in that manner, they control several processes such as cell development [48]. Like miRNAs, the dysregulation of lnc-RNAs leads to alteration in genetic expression and consequently triggering some pathologies like cancer or CNS’s disorders [49,50]. Although the mechanism of action of lnc-RNAs are not fully elucidated, different hypotheses have been raised [51].

#### Lnc-RNAs Functions

**Figure 2:**
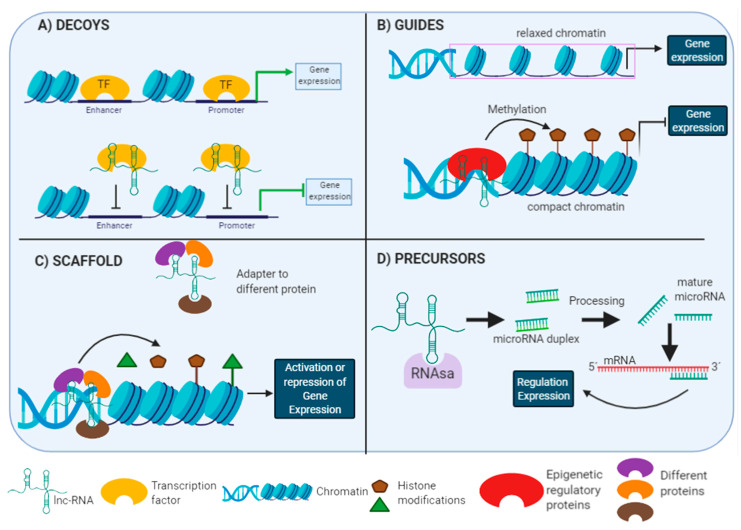
Mechanisms of action of long non-coding RNAs within a cell. Illustration of the four forms to regulate gene transcription by the lnc-RNAs. At the bottom of the figure are the conventions for each molecule that is involved in the process. (**A**) lnc-RNAs interact to DNA binding sites on transcription factors (TF). Consequently, the transcription factor binding site is inhibited and it cannot exert its function as an expression regulation. (**B**) lncRNAs direct the proteins which are involved in histone modification to particular locus. It changes the gene expression. (**C**) The lnc-RNA functions as an adapter by binding multiple effector proteins. The lnc-RNA-protein complex is directed to DNA binding sites of a specific gene leading to the regulating of its expression. (**D**) lnc-RNAs can be precursors of smaller RNA molecules such as miRNAs and they regulate the gene expression.

Lnc-RNAs present sequences similar to DNA binding sites where transcription factors (TF) are linked. In cancer, Hung et al. demonstrated that the lnc-RNA named PANDA interacts with the TF NF-YA, generating a negative feedback regulation on the expression of pro-apoptotic genes, including APAF1, BIK, FAS and LRDD [52], this happens because NF-YA cannot interact with the DNA binding sites of its target gene and subsequently an inhibition on gene expression is achieved [53] (Figure 2A). Additionally, lnc-RNAs can also be decoys for miRNAs. Studies have shown that lnc-RNAs have a high homology in the sequence with 3′UTR of mRNAs. Hence, the miRNAs can interact with lnc-RNAs instead of mRNA. As a consequence, the miRNA does not exert its function and the expression of mRNA cannot be repressed [54]. Bioinformatics analysis demonstrated that lnc-RNA SNHG1 and mRNA NLRP3 shared the same region to interact with miR-7 in Parkinson’s diseases, suggesting that SNHG1 competes with NLRP3 for binding with miR-7. Therefore, interaction between miR-7 and SNHG1 leads to elevate NLRP3 expression, resulting in NLRP3 inflammasome activation in Parkinson’s patients [55].

Lnc-RNAs can also act as guidance for proteins involved in epigenetic regulation [56,57,58,59], by recruiting proteins such as methyltransferase histones or polycomb repressor complexes, all involved in histone modification (Figure 2B). In a breast tumor model, the lnc-RNA HOTAIR binds to the PRC2, a methylase that participates in gene silencing and cancer progression [56,57]. Additionally, investigations have reported that lnc-RNAs also influence the methylation of CpG islands of DNA by recruiting DNA methylation factors, leading to gene expression. For instance, Di Ruscio et al. demonstrated in a lymphoblast model that lnc-RNA ecCEBPA interacts with methyltransferase DNMT1, where this binding prevents DNMT from methylating CEBPA’s CpG island, thus gene silencing does not occur [60].

Lnc-RNA have several domains that bind to different effector molecules (Figure 2C). The formation of the lnc-RNA-protein complex allows linking to the DNA binding sites of a particular gene leading to its activation or repression [53]. It is the case of lnc-ANRIL which functions as a scaffold to induce the binding of the WDR5 and HDAC3 proteins to form a complex. This association drives the regulation of NOX1 expression by histone modification and upregulated ROS level, among others [61].

Lnc-RNAs are precursors of smaller RNA molecules such as miRNAs (Figure 2D). For instance, it was shown that in embryonic kidney cells, the lnc-RNA H19 was the precursor to miR-675 in humans, downregulating mRNAs expressed maternally in the developing of the adjacent insulin like growth factor 2 (Igf2) gene [62], suggesting that H19 regulates gene expression through the action of miRNAs.

There are different mechanisms that lnc-RNAs employ to interact with proteins and genetic material. Thus, it can trigger an epigenetic modification and control the DNA transcription. These mechanisms demonstrate the key role that lnc-RNAs plays in the regulation of different biological processes in the organism [63]. One of these examples is the presence of lnc-RNAs in the brain. These molecules have been reported dysregulated in different brain diseases, demonstrating that lnc-RNAs could have an important role in this organ.

### 2.3. MicroRNAs and Long Non-Coding RNAs in Brain

As above mentioned, the loss and gain of non-coding RNAs functions have shown to play an important role in the regulation of multiple pathways in both normal and disease conditions [64,65,66]. Studies in brain tissues have identified that non-coding RNAs are overexpressed in CNS, where 40% of all lnc-RNAs and 50% of all miRNAs have a specific expression in the CNS and the brain, respectively [66]. Therefore, these molecules are an attractive target to delve into their functional role in the CNS.

Various researches have demonstrated that expression of specifics lnc-RNAs are associated with neurogenesis, brain development, differentiation, maturation, and neuronal activity [67,68,69]. For instance, lnc-RNAs RMST, HOTAIR1, MALAT1, Pnky or Evf-2 have been seen differentially expressed in neural cells and in processes of neurogenesis and development [70,71,72,73]. However, the mechanism of lnc-RNAs’ action over neuronal development is still unclear, but it seems like the loss function of lnc-RNAs in human embryonic stem cells may restrict neurogenesis [68]. On the other hand, in human neocortical samples from donors obtained from infancy throughout adulthood, lnc-RNAs were identified with a differential expression in an age-dependent manner. Although this study did not explore the association of these molecules to brain regulatory functions, the authors mentioned that the differential expression of these aging-dependent lnc-RNAs suggests that they may be involved in brain development and maturation [74]. In addition, there are lnc-RNAs like BC1 and MALAT1 expressed in the adult nervous system having an important role in neuronal activity and regulation of synaptic turnover [75,76]. BC1 participates in the control of neuronal excitability through the inhibition of synaptic protein postsynaptic density 95 (PSD-95) and fragile X mental retardation protein (FMRP) that are dependent on glutamate receptors (mGluR). Conversely, the absence of BC1 leads to activation of synaptic protein synthesis depending on mGluR, which triggers neuronal hyperexcitability and prolonged synchronized discharges [77]. In the case of MALAT1, investigations have shown that this lnc-RNA acts as a regulator of synaptic genes, showing that overexpression of MALAT1 leads to an increase in synaptic density, while its depletion generates the opposite effect [78].

There is strong evidence that clearly demonstrates the association between miRNA and neuronal processes. For instance, the expression of miRNAs such as miR-124, miR-125, miR-128, miR-26 and miR-29 are highly expressed in adult brain tissue which suggest that they play a differential role in each cell type [79]. Besides, it was shown that expression of these miRNAs improved response to neural differentiation in the embryonic stem cells model, suggesting they can be involved in crucial neuronal processes like development, differentiation, and proliferation [79,80]. On the contrary, miR-26 and miR-29 are present only in astrocytes, but their functions are not entirely clear [79]. Another miRNA present in adult neural stem cells is miR-137, which is involved in maturation and proliferation process, even though its expression is reduced in differentiation process [81]. On the other hand, recent evidences have shown that different miRNAs such as miR-204, miR-501, miR-223 have a pivotal role in brain process like synapse, inflammation and neuroprotection, which could trigger significant NDs [82,83,84,85,86]. Finally, some investigations demonstrated that there is a relationship between microRNAs and neuronal activity, whereby miRNAs regulate the expression of several proteins that participate in synaptic transmission, but also, neural activity can reduce the expression of microRNAs on this cells [87,88].

Each study in nc-RNAs provides information on the possible role implicated in different process in the brain. For this reason, nc-RNAs dysregulation in this organ has been associated with different brain diseases. For instance, the alteration in the expression of miR-204, miR-501, miR-34c, miR-223, miR-144 and miR-146a in the brain can contribute to accelerate cognitive decline and trigger the progression of aging and neurological disorders including Alzheimer’s disease [89]. Additionally, overexpression of nc-RNAs lead to epigenetic changes by methylation that can alter functional processes in brain and neuronal death. Consequently, several investigations have been focused in the discovery of different dysregulated nc-RNAs and their mechanisms of action in NDs development [90,91,92,93,94].

## 3. Importance of MicroRNAs and Long Non-Coding RNAs in Brain Pathologies

NDs are characterized by progressive and irreversible damage in the CNS mainly in neurons cells, causing a loss of nerve structure and function [95]. The main ND triggers is not clear yet, but some risk factors like age, inflammatory processes, obesity, viruses infection, medical condition, and genetic alterations have been associated [96,97]. Since nc-RNAs are involved in NDs, we aimed to discuss the role nc-RNAs play in NDs, in addition to making a compilation on nc-RNAs that have been reported as dysregulated in these disorders which are found in cerebrospinal fluid (CSF). In this regard, the presence in peripheral body fluids, such as CSF and serum, of molecules that could act as biomarkers for the diagnosis of NDs have become an active area of research [98,99]. Considering that, the nc-RNAs are stable molecules in the peripheral circulation, and can be detected by qRT-PCR, microarray, and sequencing, they have become a new potential class of biomarkers to be explored. In addition, understanding their involvement in disease development could depict the underlying pathogenesis of NDs, allowing new treatment approaches to be developed that act earlier in disease progression [100]. Likewise, several studies have shown that different nc-RNAs could be gene targets to modify the course of the disease [101,102], new therapeutic approaches that are focused on the modulation of multiple molecular pathway targets are necessary, because the NDs are multigenic [101].

### 3.1. MicroRNAs and Long Non-Coding RNAs as Biomarkers in Alzheimer’s Disease

Alzheimer’s disease (AD) is an age-related neurological disorder present in people over the age of 65 [103], associated with a gradual loss of neuronal synapse, synaptic functions, mitochondrial functions, and others [104]. One of its causes is the formation of aggregates of β-amyloid (Aβ) outside neurons and the deposits of abnormal Tau protein within neurons [104,105]. However, they are two out of several brain alterations that can originate the damage and destruction of neurons which causes affections in language, behavior, memory, and cognition [106,107]. Although there are different important events that are considered essential in the disease development, studies in nc-RNAs have started to explore relevant information of the AD onset, since they can regulate the expression of different proteins that can play an important role in the trigger of the disorder. It is the case of miR-26b that has shown to have a negative correlation with levels of Tau protein and the phosphorylated form Ptau in AD patients [108].

The investigators demonstrated that miR-34c regulates expression of several genes like Bcl2 and SIRT1 involved in neuronal cell survival and neuroprotection pathways, respectively [109]. Mouse models have shown a relationship between this microRNAs and a cognitive decline, because the inhibition of miR-34c rescues memory impairment in AD transgenic mice with an increase of SIRT1 expression [110]. Additionally, lnc-RNAs have also been dysregulated in AD. The expression level of lnc-RNAs BACE1-AS is increased in AD patients [111]. This molecule leads to increased expression of BACE1, a protein that participate in the generation of amyloid-β which is implicated in the pathogenesis and age-associated cognitive decline (AACD) [112]. Another lnc-RNA is EBF3-AS upregulated in the brain of AD patients [113], promotes the neuronal death by the possible stimulation of EBF3 expression, a protein which has been involved in apoptosis and cell cycle arrest in some tumor models [114]. Table 1 displays nc-RNAs dysregulated in AD, and which are present in different biofluids.

### 3.2. MicroRNAs and Long Non-Coding RNAs as Biomarkers in Parkinson’s Disease

Parkinson’s disease (PD) is the second more common ND after AD. PD is present in approximately 1% of people over the age of 60, ranging from 4.1–4.6 million people affected worldwide [145,146]. PD is characterized by bradykinesia, muscle stiffness, postural instability and involuntary tremors [147]. These signs are related to the loss of dopaminergic neurons in the *substantia nigra*, and the pathology spreads to other regions of the brain, making this disease heterogeneous an variable in progress [147]. The current diagnosis and management in patients that suffer the disease are hampered by methods with low efficiency. For this reason, PD do not have cure, and its therapy is only focused on treating the symptoms [147,148]. Some treatments include dopaminergic administration, anti-inflammatory drugs (pioglitazone), administration of gangliosides, surgical therapy, physical and occupational therapy, among others. Due to the fact that the prognosis and treatments are not very effective and timely, new therapeutic interventions such as miRNA and lnc-RNA have been investigated. These biomolecules have shown to increase the therapeutic responses and monitor the progression of the disease at early stages.

The participation of lnc-RNAs in dopaminergic neuronal death in PD has been demonstrated in vitro and in vivo approaches. The studies demonstrate that lnc-RNAs could participate in dopamine neuron differentiation, maturation and function [149,150]. Wang and coworkers demonstrated in an in vitro model that the miR-433 is crucial in the inhibition of fibroblast growth factor 20 (FGF20) [151], whose function is related to the expression of α-synuclein protein, the protein that elicits the insoluble aggregates that compose the main structure of Lewy bodies leading to the death dopaminergic neurons [152]. Another investigation was revealed that miR-7 inhibits the expression of the α-synuclein protein, making better protected against oxidative stress and Lewy body formation [153]. In a similar way, lnc-RNA MALAT1 interacted with α-synuclein protein and enhanced its stability only at the protein level but not at the mRNA level, being this was corroborated by the inhibition of this lnc-RNA reducing the expression of α-synuclein [154]. In another study, employing a model of AD in SH-SY5Y and SK-N-SH cells, it has shown that the expression of HOTAIR1 is associated to increases in pro-apoptotic caspase 3 activity, In contrast, HOTAIR1 knockdown leads to an inhibition of apoptosis, suggesting that this lnc-RNA is involved in and the progression of PD by neuronal death [92]. Other nc-RNAs dysregulated in this disorder are highlighted in Table 2.

### 3.3. MicroRNAs and Long Non-Coding RNAs as Biomarkers in Huntington’s Disease

Huntington’s disease (HD) is an autosomal dominant inherited. It means that there is a mutation in one of the two copies of the huntingtin gene (Htt) which results in the degeneration of neurons. The alteration is an expansion of the CAG triplet in HTT [170]. This gene encodes for the polyglutamine stretch in the huntingtin protein. It produces progressive damage in neurons of the cortical and striatum zone leading to their death. Due to the neuroprotective effects of the wild-type HTT protein is lost, the mutant protein originates cellular toxicity and neurological dysfunctions [171]. This leads to an exacerbated loss of neurons that has a high impact on intellectual abilities, uncontrolled movements and psychiatric disorder [172,173].

In the same way as others NDs, the treatments to Huntington’s disease are only to optimize quality of life, which includes physiotherapies, language therapies and pharmacology [174,175]. Hence, in the last year, the researchers focused on emerging therapies aimed to control molecular pathways dysregulated from the HTT gene mutation. This led to the discovery of nc-RNAs involved in this pathology which affect the expression of the different target genes associated with neuronal growth and survival [176]. For instance, one of the most recognized transcription factors (TF) in the control of neuronal development is the repressor element-1 silencing transcription factor (REST). In the normal neuronal cells, REST is hijacked in the cytoplasm by binding to wild type HTT and deprives REST from exerting its function. Therefore, in HD patients the HTT mutation cannot interact with this TF and consequently, REST translocate to the nucleus in an excessive way and subsequent decreases neuronal genes expression [176,177,178]. Investigators have demonstrated that the expression of miRNAs depend on REST [179]. It is the case of expression of miR-124a and miR-132 that have an important role in the differentiation and growth of the neuronal cells. These molecules negatively regulate the levels of hundreds of non-neuronal transcripts and enable the cell maturation and the maintenance of a neuronal phenotype [179].

However, in HD the miRNAs are repressed by nuclear REST and their downregulation result in the deprivation of neuronal identity and the neurite outgrowth [179,180]. Other nc-RNAs dysregulated in HD are the microRNA miR-9 and the lnc-RNA HAR1 whose levels are significantly lower in patients with the disorder miR-9 binds with the 3′UTR of REST mRNA, while HAR1 interacts with specific DNA regulatory motifs of REST gene. As result, these events lead in the repression of TF expression [181,182]. In addition, these nc-RNAs could be evaluated as a good candidate for the therapy, since the overexpression of these molecules in HD patients would lead to a decrease in REST by altering its activity. Likewise, the low expression of this protein would permit the maturation process, development, and adequate functioning in neuronal cells.

As mentioned, nc-RNAs are excellent candidates for regulating neuronal phenotype in HD patients which makes them valuable targets for diagnosis and treatment of the disorder. In the Table 3, shows ncRNAs reported to be dysregulated in the pathology and can affect important molecular pathways that trigger the disease. Hence, they could function as an interesting biomarker in the future making possible a rapid diagnosis and starting the suitable treatment that prolongs the lives of people with HD.

### 3.4. MicroRNAs and Long Non-Coding RNAs as Biomarkers in Amyotrophic Lateral Sclerosis’s Disease

Amyotrophic lateral sclerosis (ALS) is also a fatal neurological disorder which affects the neuromuscular function. As a result of progressive death of motor neurons in the primary motor cortex, brainstem and spinal cord, there is atrophy of the muscles that are innervated by these neurons. This leads to the death of motor neurons which is manifested as a progressive muscle paralysis of the limbs and muscles involved in speech and, in turn, swallowing and respiration are compromised, putting life at serious risk within 3–5 years of the onset of symptoms [190]. Riluzole was the first treatment approved by the U.S. Food and Drug Administration (FDA) to treat amyotrophic lateral sclerosis (ALS). It means that there is no cure for this disease, and its causes are relatively unknown. Diagnosis is based on a battery of clinical tests up to a year after symptom onset, with no robust markers of diagnosis [191]. Therefore, finding molecules that change the course of the disease is essential for the diagnostic and effective therapies in these disorders [192].

Nowadays, the majority of neurologists depend only on clinical criteria for the diagnosis of the disease. However, some studies have demonstrated that the role of nc-RNAs can be useful in both the diagnosis and treatment of ALS. Such is the case of miR-206 which is a skeletal muscle specific microRNA. The mir-206 is involved in myogenesis process, formation of neuromuscular junctions, reinnervation of denervated fibers, among other [193]. For instance, miR-206 expression delays the progression of ALS by promoting the regeneration of neuromuscular synapses according with mouse model. In addition, it was corroborated that the knockout of the microRNA in mice leads to a faster pathology development and the survival decreased significantly [194,195]. The possible mechanism of miR-206 is from suppression of HDAC4 mRNA, a regulatory factor that controls gene expression in nerves and muscles by inhibition of the reinnervation through the repression of fibroblast growth factor binding protein 1 (fgfbp1) [196,197,198].

Another upregulated miRNA in ALS is miR-338-3p which is involved in the stimulation of neuronal differentiation through negatively regulation of some genes such as MAP1A, NOVA1 and UBE2Q1 involving in the inhibition of neurite growth [199,200,201,202]. Among lnc-RNAs often deregulated in ALS is NEAT1_2, which is upregulated in spinal motor neurons at an early stage of the ALS. Although the mechanism that NEAT1_2 employs in the disorder is still unknown, it is well-known that the lnc-RNA interacts with TDP-43 and FUS/TLS proteins which are required to form paraspeckle from a set of specialized proteins and RNAs that make up this nuclear bodies in the early stage of ALS [203,204]. On the other hand, ATXN2-AS is a lnc-RNAs that is upregulated in the pathology and its presence has been associated with the neurotoxicity and may lead to ALS progression [205]. In Table 4 is highlighted other microRNAs and lnc-RNAs that have been characterized and reported as dysregulated in ALS and that could function in both the diagnosis and treatment of the illness.

Collectively, this growing body of studies shows that ncRNAs contribute to neurodegeneration in many types of dementia. Nevertheless, the majority of these studies are focused on a particular nc-RNA, and do not take into account that multiple species are functionally altered in NDs. This raises the challenge of targeting the activity of multiple ncRNAs. Although the emerging evidence have been centered in miRNAs, the most recent reports point to additional species as bioactive in mental health, mainly in the pre-symptomatic state. At the same time, the evaluation of the nc-RNAs is interesting to apply as possible treatment targets. Screening for dysregulated nc-RNAs in patients with different stages of the disease could be important in the diagnostic and evaluation of the disorder progression. For this reason, it is of great importance to implement new technologies in this branch that improve the detection of several dysregulated molecules in the pathology. In this sense, bioinformatic tools and AI have an important role in this field, since these technologies can help to compile and analyze a set of biological data, such as genes, proteins, or nc-RNAs in a specific condition from a computer [221]. Another strength of this tool is that it can study the interaction between the different molecules present inside the cell as a cell network and not as individual events, making it possible to get larger and more accurate information. Therefore, the new investigations with bioinformatic approach are aimed to predict the pathologies or detect new therapeutic targets that may be useful for treatment of the disease.

## 4. Bioinformatic Tools for the Study of Non-Coding RNAs

As discussed above, there are a vast number of miRNAs and lnc-RNAs dysregulated in NDs. Hence, in this section we illustrated different bioinformatics tools that are currently available for nc-RNAs’ data mining. Online servers provide information on the involvement of lnc-RNAs and miRNAs in various human diseases and their possible target genes and regulatory pathways. In this review, we have prioritized databases that provide information on nc-RNAs and their relationship with ND. This is made possible by information harvesting and bioinformatic predictions using the databases. On this basis, bioinformatic tools could be useful to elucidate the possible mechanism of action that nc-RNAs may have in the disorder. Therefore, the servers allow us to recognize the relevance that miRNAs and lnc-RNAs have in the development of the pathology. The Table 5 shows each available database for nc-RNAs and the corresponding reference articles, where further detailed information on the server, algorithms, and statistics of each database used for its workout can be found.

## 5. Machine Learning Applied to Diagnosis of Neurodegenerative Diseases

### 5.1. What Is Machine Learning?

The term machine learning (ML) is used to refer to AI. It consists of the development and application of computational techniques that allow a machine to mimic human intelligence [229]. However, AI acts by creating a set of rules that indicate the computer what it should do. It means that the computer only executes the rules of action for which it was configured. Consequently, it was created as subset of computer science and a technique of AI named ML that aims to develop systems that allow the computer to learn in an action-oriented manner [229,230]. To fulfill this, billions or trillions of data points (big data) must be put into the machine [231]. Subsequently, depending on the ML algorithm, the computer interprets and identifies patterns based on the set of data previously entered. Therefore, the final purpose of this tool is to generate classifications or predictions based on the information managed [232,233]. Additionally, the ML advantage is that it allows computer algorithms to mechanize data and learn through experience, without being programmed. In other words, the model improves in-dependent manner, making the system more robust over time [233,234]. Currently, ML employs two types of learning: supervised learning and unsupervised learning.

**Figure 3:**
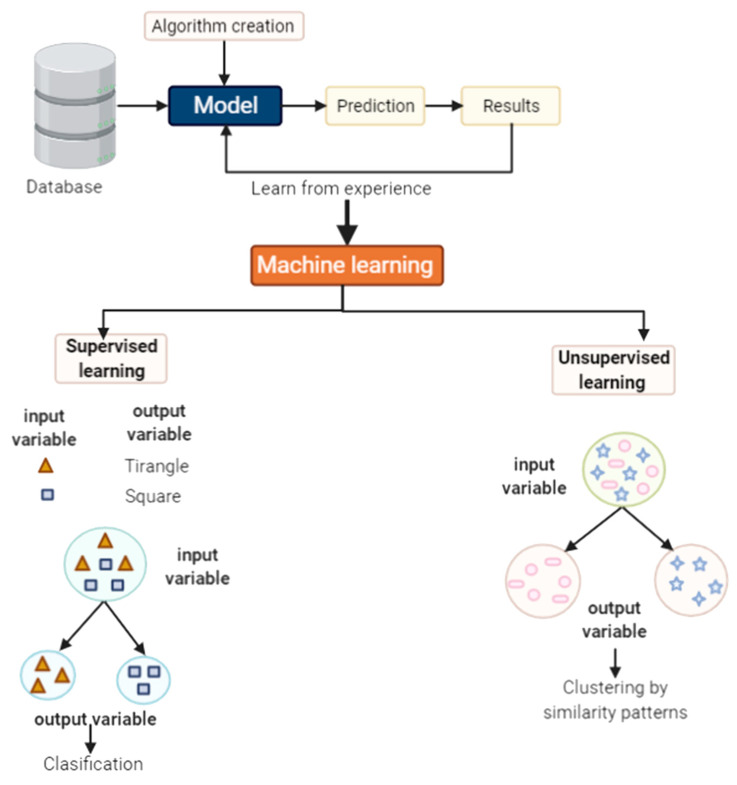
Supervised and unsupervised learning. Illustration for creating a model of machine learning according to the data set and the objective of the investigation.

Supervised learning consists of showing pre-classified data to the machine, making it capable of classifying unknown information based on previews data. It means that known input variables are entered into the computer, alongside with their attribute descriptions (data and label) which are later related to output variables. All of these data are employed to teach the machine. After training, the new data can be entered without a label to be sorted and labeled by the machine, on the basis of the patterns that have been recorded during the training [234,235] (Figure 3). In the case of medicine, supervised learning is mainly used in the automated interpretation of some diagnoses by identifying certain labeled data that were previously created and associated with disease.

Unsupervised learning is based on the classification of data set based on similar patterns among them [235]. To cluster data, algorithms are needed to identify similar features in the information, according to the requirement of the study [235]. The integration of unsupervised learning in medicine has been used to correlate the patient with a diagnosis [236]. For instance, a data set could be obtained from a specific examination in a wide group of patients with some pathology. Information can be collected from biopsies, diagnostic images, or gene expression microarrays, among others. And then, through the implementation of unsupervised machine learning algorithms, patterns in the data can be determined. This will lead to classification of the patients into a diagnostic group that depends on their pattern (Figure 3). Thus, unsupervised learning could guide the therapies to be used based on the classification [237,238]. It should be noted that, unlike supervised learning, there is no expected outcome, but rather a search for patterns in the data [21].

### 5.2. Some Methods Used in Machine Learning

One of the most important parts when employing ML is the model selection. The model defines the type of relationships between the input and the output, and data can be classified depending on the strategy of learning. Regardless of whether it is supervised or unsupervised learning, each one of them has particular characteristics, making it necessary to take several aspects into consideration [235,239]. The model development can be summarized in the following four steps:

(a) Data collection and processing: Data transformation, normalization and optimization.
(b) Model selection: Selection of suitable algorithms and choice of a success indicator.
(c) Model training: Precision, accuracy, sensitive and specificity.
(d) Model validation: Parameter setting and reproducibility.

#### 5.2.1. Supervised Learning

- Regression

In this type of supervised learning, the purpose is to estimate the output value from a set of input values, thereby this model is used for estimating values such as weigh, price and length, among others. A supervisor is required for the training to provide the desired output and to be able to predict the value of the model based on the above inputs with their respective desired outputs. The simplest method of this type is linear regression, which assumes a linear relationship between an independent input variable (X) and dependent output variable (Y). The main scope is to determinate a value (dependent variables) according to the prediction of the independent variables. This is employed to prognostic cause and effect between the variables (X, Y). Therefore, this algorithm is very useful in medicine in prediction or prognostic of some diseases [240,241].

- Classification

It consists of creating some classes from a set of data information (training set) according to established patterns. The goal is that the model can automatically classify a new sample into one of these groups bearing in mind the established patterns from the training set. This type of method may be used to classify a tumor as benign/malignant/healthy [241]. Some algorithms of this type are Decision Trees, Random Forests, Support Vector Machine, K-Nearest-Neighbor (KNN), among other. In this regard, a trained model can make a prediction based on the training set, where the model outputs the probability that: (i) a class member has a certain attribute, (ii) an arbitrary data point belongs to that class and (iii) an arbitrary data point has a specific characteristic. One example of this type is Bayesian algorithms, which are based on the Bayes’ theorem. It is the simplest and most powerful probabilistic classifier to use on a large data set. Bayesian algorithms use probability to predict a class or category in the function of the input variables. The model assumes that all the variables introduced into the model are independent. This means that the presence of a certain feature in a data set is not related to the presence of some other feature [235,242,243]. Finally, Artificial Neural Networks (ANN) is another type of classifier, whose is derived from the similarity with the signaling behavior of the neurons in the biological neural network. Neural networks are useful in the evaluation of complex interactions between a group of diverse measurable variables that ultimately lead to the prediction of an outcome [244,245]. ANN are made up of layers of neurons:

(a) An input layer corresponds to independent variables. The model is trained by inputting information through this layer.
(b) One or more intermediate layers or hidden layers communicated with each other through activation functions. The information is processed within this layer.
(c) An output layer corresponds to dependent variables. The predictions are given through this layer.

#### 5.2.2. Unsupervised Learning

- Clustering

In the unsupervised learning approach, there is no desired output known, making this type of algorithms tries to find clusters or groups in the unlabeled data based on their similarity. Thus, the elements of the same group present similar characteristics to each other and different to the other groups. For instance, k-means algorithm groups the input data into the number of clusters (k), in which the desired number of the independent input variables should be obtained, and it is determined at the beginning of the model development. Subsequently, data points are randomly fixed depending on K. In other words, if K = 2 is desired, the randomly selected points will also be 2. These points will be called centroids and they define the clusters with respect to the proximity between the data and the centroid. Data close to a certain centroid, suggest that these data have similar characteristics and thus the group of that centroid will be formed. These analyses are repeated several times, fixing different centroids until they begin to have similar results consecutively [246].

Another is dimensionality reduction, which is a statistical procedure that attempts to reduce the number of the initial data (original variables). This method is applied to a big data set with a greater number of possibly correlated quantitative variables, suggesting that there is redundant information. Therefore, the reduction is made in order to improve data processing, yet covering as much information as possible. This type of algorithm reduces the original variables to a lower number of transformed variables. Besides, these data are characterized by being independent or uncorrelated to each other, as well as data organized according to their information [247,248,249]. The sub-types of these method are Principal Component Analysis (PCA), Linear Discriminant Analysis (LDA), Factorial Analysis (FA), Multidimensional Scale (MDS), Generalized Discriminant Analysis (GDA), among other.

- Association

Although it is also known as association rules mining, it is a type of ML focusing on finding relationships and dependencies among variables in large data sets, which is consider essential for extracting knowledge from data [250]. In short, each method has benefits and difficulties that are important to keep in mind. Table 6 shows some advantages and disadvantages of methods of ML described above that may be practical at the time of choosing and developing the model that best fits the problem presented.

A common issue with all the ncRNA characterization models is finding the right balance of specificity and sensitivity in order to yield better accuracy, due to all these algorithms suffer from type I error false positive. It is suggested that a better combination of sequence-intrinsic and non-species-specific features, deep learning models, and training on larger datasets might address the challenges of accuracy for the classification of ncRNA species. Besides, it is important to highlight that is difficult to determine which of the methods is better than the other or which one has the best answer, since it depends on the problem to be studied. However, each one has some conditions and features that can be useful at the time of selecting the correct method considering the problem and the purpose, and thus fitting the parameters and the variables to the type of model chosen.

### 5.3. Machine Learning in Brain

#### Machine Learning as a Diagnostic Tool of Neurodegenerative Diseases from MicroRNAs and Long Non-Coding RNAs

Currently, there are several studies showing a high or low expression of miRNAs and lnc-RNAs using biofluids in patients with some type of ND, in comparison with healthy people [251]. As described in the second section, the presence of nc-RNAs in biofluids is a great interest in the study of pathology, due to the ease of obtaining such samples. By this way, the differential expression of nc-RNAs in biofluids between patients with ND and healthy people can be used for the development and training of ML models in order to diagnose these disorders quickly and accurately. For instance, Ludwing et al. conducted a study selected the top miRNAs that were associated with AD in literature. A final set of 21 miRNAs was selected. Among them, 18 miRNAs were significantly correlated with neurodegeneration using by qPCR in samples of blood among AD patients compared to three groups: mild cognitive impairment, other neurological diseases, and healthy controls. As a result, the miRNAs were used as the input data to train different ML algorithms, where the boosted tree was the model with best result for the AD classification and the other three groups. This model reached an area under the curve (AUC) value of 87.6% in differentiating AD patients from controls and 10 miRNAs were validated, in particular miR-26a/26b-5p a well-studied brain-specific miRNA in synaptic function [14].

Another research collected blood samples from 48 AD patients, and 22 healthy controls and next-generation sequencing was used to evaluate the differential expression of such samples, showing that 140 miRNAs had a significant change in expression levels. Among these, 12 miRNAs involved in CNS development were selected, and used as inputs for a ML model construction. The model was able to classify between AD patients and controls, obtaining an accuracy of 93.3%. In addition, this ML model was also applied to treat AD patients and other types of NDs (PD, multiple sclerosis, mild cognitive impairment), being AD the worst classified with 73% and 82.8% for controls and other NDs. The majority of 12-miRNA signature have not been identified or investigated so far in relationship to AD [252].

Regarding the evaluation of lnc-RNA expression profiles, a study assesses this by using data from public available Gene Expression Omnibus (GEO). From which 57 AD samples and 57 healthy people (controls) were selected, the authors found 47 lnc-RNAs differentially expressed. Yet, a panel of 9 lnc-RNAs strongly involved in brain development were ultimately applied to the train a ML model in the 114 samples. A supervised method was used to construct the lncRNA-based classifier for the classification of AD and healthy control samples. The test achieved a classification of 49 patients out of 57 with AD and 51 out of 57 healthy control samples. The model had a predictive power accuracy of 87.7%. Furthermore, to assess the robustness, the model was evaluated with more independent samples, where it was able to classify 79 of 87 AD patient samples, and 62 of 74 healthy control samples, which means a predictive accuracy of 87.6%. The LncSigAD9 was able to differentiate between AD and healthy controls with high diagnostic sensitivity and specificity both in the discovery cohort (86.3 and 89.5%) and the additional independent AD cohort (90.8 and 83.8%) [143]. These scientific gains highlight the importance of lncRNAs in brain aging and AD, which can serve as promising biomarkers for the diagnosis and treatment of AD at earlier stages. In the Table 7, describes other studies that have shown the use of lnc-RNAs and miRNAs as biomarkers. We illustrate the different methods of ML to detect the types of NDs based on the expression profile of nc-RNAs and the accuracy of the experimental, and among others.

These studies represent the important role that ML has recently emerged in the detection of the different types of NDs. Although most ML studies have used protein-coding genes to detect the diseases, nc-RNAs could also be suitable candidate as predictive biomarkers for AD detection, since they are stable molecules in different biofluids and could be detected at early stages for NDs. In addition, the process to obtain the molecules is minimally invasive. Therefore, these investigations show a breakthrough about the approach in the diagnosis of different types of NDs, even more, when current mechanisms of detection have a low accuracy.

Although the use of ML to aid diagnosis, prognosis and therapeutic development is still in its infancy, this technology might in the future enable more precise, earlier diagnosis of NDs on the basis of medical history and through the identification of more specific diagnostic of nc-RNAs biomarkers. Further studies should assess the ability of the ML-based predictive models applied to ND data, which will require selecting the right datasets for training and validation and knowing how to deal with missing data. This will provide a more accurate diagnosis that could be followed by a personalized treatment regimen according to the nature of each NDs.

## 6. Conclusions

In this review we overview several nc-RNAs involved in neurological processes and NDs. Some researchers have even shown that dysregulation of these molecules may be measured from biofluids, which makes nc-RNA a promising biomarker candidate for detecting NDs. In this regard, ML plays an outstanding role, since it can easily solve the main concern in this respect, dealing with the vast amount of nc-RNAs that need to be analyzed. Upon closer inspection of the current analysis of nc-RNAs with ML, this approach shows fair accuracy by classifying healthy and disease samples, meaning that differentiation patter is very clear in those scenarios. Yet, the specificity drops when the models try to discriminate among different NDs, which is also common to the current diagnostic methods that are based on clinical symptoms. Therefore, future work should be focused on improving the chances of the ML models to identify the unique patter of each disease. The latter may be performed, either by collecting more data samples to train the model, by building models that can find smaller patterns from the training data or rather a combination of both. For this purpose, caution should be taken in timing of treatment, selection of the patients, and the lack of multifactor approach, since large inter-individual variability is seen among patients and control which it could lead bias in the outcomes. In addition, it is suggested to understand that the functional interaction between different classes of nc-RNAs provide an additional layer of complexity, in which multiple ncRNA-signaling pathways are involved. Still, we may experience a significant increase in the use of nc-RNAs, and it will help towards achieving more personalized treatments and effective precision medicine in the foreseeable future.

## Figures and Tables

**Table 1:** Dysregulated nc-RNAs in human samples of Alzheimer’s disease.

Source	Target Genes	Condition	Dysregulated nc-RNAs	Study
Whole-blood		1	miR-26b-3p, miR-28-3p, miR-30c-5p, miR-30d-5p, miR-148b-5p, miR-151a-3p, miR-186-5p, miR-425-5p, miR-550a-5p, miR-1468, miR-4781-3p, miR-5001-3p, and miR-6513-3p.	[115]
		2	let-7a-5p, let-7e-5p, let-7f-5p, let-7g-5p, miR-15a-5p, miR-17-3p, miR-29b-3p, miR-98–5p, miR-144-5p, miR-148a-3p, miR-502-3p, miR-660-5p, miR-1294, and miR-3200-3p.	
		2	miR-29b, miR-107, miR-125b, miR-146a, miR-181c, and miR-342	[116]
		1	BACE1-AS	[117]
PBMCs		1	miR-34a, miR-34b, miR-34c and miR-181b	[118]
Serum		2 1	miR-23a, miR-26b, miR-125b and miR-181c. miR-9	[119]
		2	miR- 98-5p, miR-191-5p, miR-342-3p, miR-483-3p, miR-885-5p, and let-7d-5p	[120]
Serum exosomal		1	miR-15a-5p, miR18b-5b, miR-20a-5p, miR-30e-5p, miR-93-5p, miR-101-3p, miR106a-5p, miR-106b-5p, miR-143-3p, miR335-5p, miR-361-5p, miR-425-5p, miR-582-5p and miR-3065-5p	[121]
		2	miR-15b-3p, miR-342-3p and miR-1306-5p	
Plasma	BCL2, SIRT1	1	miR-34c	[122]
		2	let-7d-5p, let-7g-5p, miR-15b-5p, miR-142-3p, miR-191- 5p, miR-301a-3p, and miR-545-3p	[122]
Plasma exosomal		3	miR-23b-3p, miR-24-3p, miR-29b-3p, miR-125b-5p, miR-138-5p, miR-139-5p, miR-141-3p, miR-150-5p, miR-152-3p, miR-185-5p, miR-338-3p, miR342-3p, miR-342-5p, miR-548at-5p, miR-659-5p, miR-3065-5p, miR-3613-3p, miR-3916, miR-4772-3p and miR-5001-3p	[123]
Cerebrospinal fluid (CSF)		3	miR-100, miR-103, miR-146a, miR-219, miR-296, miR-335, miR-375, miR-449, miR-505, miR-708, miR-766, miR-1274a, miR-3622b-3p, miR-4467 and miR-4674	[124]
		2	miR-10a, miR-10b, miR-15b, miR-99a, miR-124, miR-125, miR-126, miR-127, miR-142-5p, miR-143, miR-146b, miR-154, miR-181a, miR-181c, miR-194, miR-195, miR-199a, miR-221, miR-328, miR-422b, miR-451, miR-455 and miR-497	[125]
		1	miR-9, miR-125b, miR-146a and miR-155	[126,127]
		1	Let-7f, miR-30a-3p, miR-30a-5p, miR-30b, miR-30c, miR-30d, miR-32, miR-105, miR-125a, miR-135a, miR-138, miR-141, miR-151, miR-186, miR-191, miR-197, miR-204, miR-205, miR-216, miR-302b miR-345, miR-362, miR-371, miR-374, miR-375, miR-380-3p, miR-429, miR-448, miR-449, miR-494, miR-501, miR-517, miR-517b, miR-518b, miR-518f, miR-520a and miR-526a	[125]
		2	MALAT1	[128]
Brain	SPT	1	miR-9, miR-29a, miR-29b-1, miR-15, miR-137 and miR-181c	[129]
	SPTLC1	1	(miR-181c y miR-137) *	
	Secretases	1	miR-146	[130]
	BACE1/ SPTLC2	2	miR-9, miR-29a, miR-29b-1 and miR-124 *	[131,132]
	TAU	1	miR-26b and miR-34a	[133,134]
	APP	4	miR-101, miR-106a, and miR-520c	[135,136]
	APP	2	miR-124 *	[137]
	BACE1 APP	3	miR-15a, miR-29b-1, miR-9, and miR-19b, let-7, miR-101, miR-15a, and miR-106b	[138]
	IGF-1	4	miR-98 *	[139]
		1	XIST, LNC01094, NEAT1, VAC14-A81, lnc-SERF1B-1, RP11-274-H2-5, AF001548-5, LINC00844, lnc-VS1G10-1, lnc-POTEG-4, EMX2OS, lnc-INADL-2, lncXRN2-2, RP11-953820-1, lnc-ADAM30-1, LINC00320 and lnc-TAF9-2	[140]
	miR-15/107	1	NEAT1 (In temporal cortex and hippocampus), HOTAIR (In hippocampus and cerebellum)	[141]
	BACE1	1	BACE1-AS	[142]
	SORL1		51A	
	GABA B		17A	
	BACE and γ-secretase		NDM29	
	eIF4A		BC200	
			BDNF-AS SOX2OT	
	Rad18		NAD-RAT 18	
		3	MIR7-3HG, AL109615.3, NEBL-AS1, ATP6V0E2-AS1, PDXDC2P-NPIPB14P, LOC441204, A2M-AS1, TGFB2-OT1, LINC00672 and LncSigAD9	[143]
		1	n336934	[144]
		2	n341006	

1. Upregulated in AD patients; 2. Down regulated in AD patients; 3. Dysregulated expression in AD patients; 4. Overexpression in AD model cells; * Determination in in vitro assays or using model animals.

**Table 2:** Dysregulated nc-RNAs in human samples of Parkinson’s disease.

Source	Target Genes	Condition	Dysregulated nc-RNAs	Study
Blood		2	miR-1, miR-22p and miR-29a	[155]
		1	miR-16-2-3p, miR-26a-2-3p and miR-30a	
		1	miR-18b, miR-20a miR-21, miR-30b, miR-103a, miR-150, miR-199b, miR-378c, miR-1274b, miR-671, miR-1249. miR-4293	[156]
		2	miR-1, miR-16, miR 22, miR 29a, miR-92b, miR-320a, miR-320b, miR-320c, miR-769	
		1	LINC00302 and LINC00328	[157]
		2	FAM215A, MCF2L-AS1, NOP14-AS1, PART1, XIST	
		1	AC131056.3-001, HOTAIRM1, lnc-MOK-6:1, and RF01976.1-201	[158]
		1	TM4SF19-TCTEXID2, LOC101927369, LOCI102724104, LINC01871, LOC105373420, LOC105371464, LINC00943, LOC105370060, LOC101927012, LOC105372055	[159]
		2	LOC102724765, LOC105369772, KRT73-AS1, LOC105379392, JHDM1D-ASI, LOC105372185, LOC105377225, LOC105378701, LOC105375056 and LOC105373204	
PMBCs		2	miR-19b, miR-26a, miR-28-5p, miR-29b, miR-29c, miR-30b, miR-30c, miR-126, miR-151-3p, miR-147, miR-151-5p, miR-199a-3p, miR-199a-5p, miR-199b-3p, miR-301a, miR-335, miR-374a, miR-374b	[156]
Plasma		1	miR-181c, miR-331-5p, miR-193a-5p, miR196b, miR-454, miR-125a-3p and miR-137	[160]
		2	miR-222, miR-505, miR 626	[156]
Serum		2	miR141, miR-214, miR-146b-5p and miR193a-3p	[161]
		1	miR-233, miR324-3p and miR-24	[162]
		2	miR-339-5p, miR-30c and miR-148-b	
		1	miR-24, miR 30a-3p, miR-30e-3p, miR-195, miR-223, miR-324-3p, miR-338-3p	[156]
		2	miR-15b, miR-16-2-3p, miR 19b, miR-29a, miR 29c, miR-30c, miR 148b, miR-181a, miR-185, miR-221, miR 1294	
CSF		1	miR 10a-5p, miR19a-3p, miR-16-2, miR19b-3p, miR-26 miR-30b, miR-103a, miR-127-3p, miR-132-5p, miR 136-3p, miR-153, miR-331-5p, miR-370, miR 485-5p, let-7g-3p, miR-409-3p, miR-433, miR 873-3p	[156]
		2	miR 1, miR 19b-3p, miR 19c, miR 22, miR-28, miR 29, miR 119a miR-128, miR 132-5p, miR-126, miR-127-3p, miR-151, miR-212-3p, miR-301a, miR-370, miR 374, miR-409-3p, miR-485-5p, miR-873-3p, miR-1224-5p, miR-4448	
Brain		2	miR 19a, miR-19b, miR-29a and miR-29c	[163]
	SNCA	1	miR-7 and miR-153	[126]
	E_2_F_1_	1	miR-184	[164]
	LRKK2	1	miR-205	[165,166]
	DP	1	let-7	[162]
	FGF20	1	miR-433	[167,168]
		1	miR-16-5p, miR 21, miR26b, miR-29a-3p, miR 106a, miR 127-5p, miR 224, miR 301b, miR 373, miR 548d	[143]
		2	miR 10b-5p, miR 22, mir 29a, miR 29b, miR 29c, miR 127-3p, miR 135b, miR 181a, miR 181b, miR181c, miR 181d, miR 184, miR 198, miR 205, miR 485-5p, let-7i-3p/5p, miR 1224	
	Sox2	1	SoX2OT	[169]
	PINK 1		naPINK1	
	PINK1 UCHL1	3	BC200 PINK1-AS UCHL1-AS	
	SNCA	2	SNCA-AS1	[150]
	LRRK2		AK127687	
	UCHL1		UCHL1-AS1	
	PINK1		PINK1-AS1	
	DJ1		AX747125	
	MAPT		MAPT-AS1	

1. Upregulated in Parkinson patients; 2. Down regulated in Parkinson patients; 3. Dysregulated expression in Parkinson patients.

**Table 3:** Dysregulated miRNAs in human samples of Huntington’s disease.

Source	Target Genes	Condition	Dysregulated miRNAs	Study
Plasma		1	miR-22-5p, miR-30d-5p, miR-128, miR-130b-3p, miR-223-3p, miR-223-5p, miR-222-3p, miR-338-3p, miR-361-5, miR-425-5p, miR-628-3p, miR-877-5p and miR-942	[183]
Parietal cortical tissue	REST	1	miR-29a and miR-135b	[176]
		2	miR-132	
Brodmann’s area 4 (BA4)	REST	2	miR-9, miR-29b and miR-124a	[181]
		1	miR-132, miR-486 and miR-196a	
Frontal cortex and striatum		1	miR 15a, miR-15b, miR-16, miR-17, miR-19b, miR 20a, miR 27b, miR-30a, miR-30b, miR-30c, miR-30e, miR-33a, miR-33b, miR-92a, miR-93, miR-99b, miR-100, miR 101, miR 106b, miR-126, miR-145, miR-146a, miR-148b, miR-151-5p miR-151-3p, miR-181a, miR-193b, miR-199b-3p, miR-204, miR-219-2-3p, miR-219-5p, miR-338-3p, miR-363, miR-451, miR-486-5p, miR-887, miR1250, miR-1974	[184]
		2	miR-95, miR-103, miR-107, miR-124, miR-127-3p, miR-128, miR-139-3p, miR-181d, miR-221, miR-222, miR-323-3p, miR-330-3p, miR-369-5p, miR-382, miR-383, miR-409-5p, miR-423-5p, miR-432, miR-433 and miR-483-3p, miR-485-3p, miR-485-5p, miR-495, miR-543, miR-598, miR-708, miR-1224-5p, miR-1301, miR-1307	
Brain	HTTA PCR2	2	HTT-AS MEG3 HAR1R HAR1F	[169,185]
		1	TUG1, LINC00341, RPS20P22 and NEAT1	[186,187]
		2	MEG3, DGCR5 and LINC00342	
	BDNF HTT	3	BDNF-AS HTT-AS	[188,189]

1. Upregulated in Huntington patients; 2. Down regulated in Huntington patients; 3. Dysregulated expression in Huntington patients.

**Table 4:** Dysregulated nc-RNAs in human samples of Amyotrophic Lateral Sclerosis disease.

Source	Target Genes	Condition	Dysregulated nc-RNAs	Study
Serum	HDAC4	1	miR-206 miR 106b	[190]
		2	miR-4747.5p, miR-3665, miR 1915-3p, miR 4530	[206]
		2	miR 132-5p, miR 132-3p, miR 143-5p, miR-143,3p, miR and LET7B-5p	[207]
		2	miR-1234-3p and miR-1825	[208]
		1	miR-143-3p, miR-206	[194]
		2	miR-374b-5p	
		1	miR 142-3p	[209]
		2	miR 1249-3p	
Plasma	EPHA4	1	miR-4649-5	[210]
		2	miR-4299	
		1	miR-4258 and miR-663b.	
		2	miR-26b-5p, miR-4299, let-7f-5p, miR-4419a, miR-3187-5p and miR-4496	
		1	miR-424 and miR 206	[195]
		1	miR-206, miR-338-3p, miR-9, miR-129-3p and miR-335-5p	[211]
Whole Blood		1	miR-338-3p	[212]
		2	miR-451, miR-1275, miR-328, miR-638, miR-149, miR-665, miR-583	
		2	let-7a-5p, let-7d-5p, let-7f-5p, let-7g-5p, let-7i-5p, miR-103a-3p, miR-106b-3p, miR-128-3p, miR-130a-3p, miR-130b-3p, miR-144-5p, miR-148a-3p, miR-148b-3p, miR-15a-5p, miR-15b-5p, miR-151a-5p, miR-151b, miR-16-5p, miR-182-5p, miR-183-5p, miR-186-5p, miR-22-3p, miR-221-3p, miR-223-3p, miR-23a-3p, miR-26a-5p, miR-26b-5p, miR-27b-3p, miR-28-3p, miR-30b-5p, miR-30c-5p, miR-342-3p, miR-425-5p, miR-451a, miR-532-5p, miR-550a-3p, miR-584-5p, miR-93-5p	[213]
		1	miR-34a, miR-100, miR-193b and miR 4485	[214]
		2	miR-124, miR-183, miR-193b, miR-451, miR- 3690, miR-3935, miR-4538, miR-4701	
Cerebrospinal Fluid (CSF)		1	miR-143-5p and miR-574-5p	[207]
		2	miR-132-3p, miR-132-5p and miR-143-3p	
		1	miR 181a-5p	[215]
		2	LET7A-5p, LET7B-5p, LET7F-5p, miR-15b-5p, miR-21-5p, miR-195-5p, miR-148-3p	
Skeletal muscle	PGC-1α	1	miRNAs-23a miR-29b, miR 31, miR-206 and miR-455	[216]
Substanzia nigra		1	miR-26b	[134]
Spinal cord	NFL	1	miR-16-2, miR-508-5p, miR 558	[217]
		1	miR-146	
		2	miR-524-5 miR 582-3p	
		2	miR-23a, miR-23b, miR 30a, miR-30b, miR-192, miR-193a-5p, miR-215, miR 520e, 548a-5p, miR 556-5p, miR-606, miR-612, miR-624, miR-647 and	
		1	miR-24-2, miR-142-3p, miR-142-5p, miR-146a, miR-146b, miR-155	[218]
		2	miR-148b-5p, miR- 577, miR 133b and miR-140-3p	[219]
	NEFL	2	miR-b1336 and miR-b2403	[220]
Brain	NAV3	1	miR 29a miR 29b and miR-338-3p	[199]
	NEFL	1	miR-9.	[198]

1. Upregulated in ALS patients; 2. Down regulated in ALS patients; 3. Dysregulated expression in ALS patients.

**Table 5:** Databases of non-coding RNAs and their involvement to diseases. Different online platforms are described in which are possible to search microRNAs or long non-coding RNAs of interest and their participation with different diseases like NDs. The data reported in these servers have been mostly experimentally corroborated or are reported by bioinformatic predictions.

Database	Platform Functions	Website	Reference
**LncRNA and disease database**	Database makes association between lnc-RNAs and different diseases supported obtained experimentally or by predictions. LncRNADisease also cures lnc-RNA interactions at other levels, including proteins, RNA, miRNA and DNA, which leads to suggesting possible mechanisms of action.	http://www.cuilab.cn/lncrnadisease (Accessed on 18 February 2021). http://www.rnanut.net/lncrnadisease/ (Accessed on 22 June 2021)	[222,223]
**LncTard**	LncTarD offers information on the actions of lnc-RNAs and miRNAs in human diseases. The platform shows the gene that is being regulated by non-coding RNAs. The regulation mechanism and the influence that these molecules have on cell function.	http://biocc.hrbmu.edu.cn/LncTarD/jsp/Browser.jsp (Accessed on 18 February 2021).	[224]
**LncBook**	The server presents information about human lncRNAs along with multi-omics data integration and their association with various diseases. The functional mechanism of lncRNAs is associated for each pathology, as well as their role in the disease, among other characteristics.	http://bigd.big.ac.cn/lncbook/index (Accessed on 19 February 2021).	[225]
**The Human microRNA Disease Database (HMDD v3.0)**	Database presents the links between microRNA-disease supported experimentally. HMDD v3.0 presents information about the functional enrichment of each miRNA with its target gene and online visualization of their interaction network.	http://www.cuilab.cn/hmdd (Accessed on 18 February 2021).	[226]
**miRNA SNP Disease database (MSDD)**	MSDD integrates experimentally reported data from miRNAs with single nucleotide polymorphisms (SNPs) and their involvement in a disease. Each miRSNP-disease association shows information about miRNAs, SNPs, the miRNA target gene(s) and disease names, further a brief functional description.	http://bio-bigdata.hrbmu.edu.cn/msdd/browse.jsp (Accessed on 19 February 2021).	[227]
**miRwayDB**	miRwayDB shows the relations of experimentally validated microRNA pathways in several diseases. The platform contains information about the disease condition, the associated miRNAs, the types of experimental samples, the regulation pattern (up/down).	http://diana.imis.athena-innovation.gr/DianaTools/index.php?r=mirpath/index (Accessed on 19 February 2021).	[228]

**Table 6:** Methods of Machine learning. Brief description of different algorithms mostly employed in both supervised and unsupervised learning in scientific investigations.

Method	Features	Advantage	Disadvantage
	**Supervised Learning**		
Linear regression	There should be a relationship between the dependent and independent variable. The model can be adjusted to the data when the input variables are highly correlated. The model is going to be more accurate if the variables have a normal distribution.	It is easy and fast to model. It has less possibility to over-adjustment. It is useful when there is numerical data with many features. The data size is not a problem.	It is not useful for complex models. In this method, there cannot be non-linear relationships without transforming the input data. It can suffer with atypical values. The method assumes that the data are always linear.
Method of classification	It is necessary to evaluate the participation of each node. There must be a terminal node and a class should be assigned to determine the classification. Non-significant predictor variables are grouped together to form combined categories and that way they are meaningful.	The training is simple These methods are powerful and accurate. The methods control lost values. It is possible to model complex relationship non-linear.	The training can be slow if the data size is big. The parameters of model are sometimes difficult to interpret. They can suffer over-adjust.
Bayesian Algorithms	The method is based on the supposition that the unknowns of interest follow probabilistic distributions. Bayesian algorithms allows determinate of the probability of occurrence of a hypothesis of quantitative way. The data of training can affect the probability of the hypotheses. In the method can include a priori knowledge like the probability of each hypothesis.	This is easy and fast to implement. It does not require much memory. It is easy to interpret. It can be model complex systems. It is accurate when the data size is small.	It suffers from irrelevant characteristics. Bayesian algorithms fails to estimate rare characteristics. It is necessary to have a priori knowledge. The method has a high computational cost.
Artificial neural networks (ANN)	Number of layers is related with changes in network structure and then modify the results. Number of neurons per layer can modify the efficiency of learning. Degree of connectivity between neurons. A high number of interactions can make the training slower. Type of connection between neurons. According to these conditions the training and the function of neuronal networks will vary.	ANN creates its own representation of the information. It has tolerance to fail and a non-significant change in the input data. ANNs can operate in real time. They can develop their tasks in different computers in parallel.	It is difficult to teach an ANN complex task. The teaching is slow and needs a long time. The method requires a big data for the training. ANNs do not allow interpret what they have been learned. It means that requires the intervention of the programmer.
	**Unsupervised learning**		
K-means clustering	The quality of the clustering method depends on similarity measure. Clustering assumes that each instance belongs to exactly one cluster. The data are reassigned to clusters based on their distance from the centroids.	It is easy to understand and adapt. This works well with large or small data sets. The method is efficient, powerful and performs well.	The user must define the number of clusters (K) in advance. The method is sensitive to the noise. The result may vary based on the clusters chosen at the beginning.
Dimensionality reduction	This method is useful when the transformation of variables is not possible. The model eliminates variables that do not offer a new information in data set. The model creates a new variable from old variables.	The method allows eliminate irrelevant and redundant variables which improves computer performance what reduce the time, the storage space and the costs. Dimensionality reduction allows diminish the complexity which facilitates to understand the model and its results. The elimination of multicollinearity improves the performance of the machine learning model. In this method is easier to visualize data when reduced to very low dimensions such as 2D or 3D.	Possible loss of information by variables eliminated that have a connection with someone important variable. Subtypes like PCA tends to find linear correlations between variables, which is sometimes unfavorable. PCA fails in cases where covariance and mean are not enough to define datasets.

**Table 7:** Machine Learning to detect neurodegenerative diseases from non-coding RNAs. The table describes the main results in the diagnosis of NDs from studies reported in the literature and the accuracy in the algorithms of Machine Learning that were employed to classify patients with NDs and healthy controls.

Year	ML Method	nc-RNAs Included	Dysregulated nc-RNAs	Biofluid Sample	Technique Used	Patients	Higher Accuracy	Study
2019	Supervised learning: Decision trees (Boosted tree model)	21 miRNAs	18 dysregulated miRNAs	Blood	qPCR	Patients with AD in comparison with three groups: mild cognitive impairment, other neurological diseases, and healthy controls	83.5%	[14]
2018	Supervised learning: Biomarker Optimization Software System (BOSS)	15 miRNAs	5 dysregulated miRNAs	Cerebrospinal Fluid	Next-generation sequencing	40 patients with early-stage PD vs. 40 healthy controls	82%	[250]
2015	Supervised learning: Support Vector Machines (SVM) Decision trees model (J48, adaboostM1)	20 miRNAs	7 dysregulated miRNAs	Plasma sample	Deep sequencing	35 patients with AD vs. 35 healthy people	83–89%	[123]
2013	Supervised learning: Support Vector Machines (SVM)	140 miRNAs	12 dysregulated miRNAs	Blood sample	Next-generation sequencing	48 patients with AD vs. 22 healthy controls	93.3%	[252]
2018	Supervised learning: Support Vector Machines (SVM)	47 Lnc-RNAs	9 dysregulated Lnc-RNAs	Data available in GEO.	Microarray	57 patients with AD vs. 57 healthy people (controls) from the platform	87.7%	[143]

## Data Availability

Not applicable.

## References

[1] Tsagalioti E., Trifonos C., Morari A., Vadikolias K., Giaginis C. (2016). Clinical value of nutritional status in neurodegenerative diseases: What is its impact and how it affects disease progression and management?. Nutr. Neurosci..

[2] Chen W.-W., Zhang X., Huang W.-J. (2016). Role of neuroinflammation in neurodegenerative diseases (Review). Mol. Med. Rep..

[3] Mitchell K.J. (2011). The genetics of neurodevelopmental disease. Curr. Opin. Neurobiol..

[4] Gómez-Río M., Caballero M.M., Górriz Sáez J.M., Mínguez-Castellanos A. (2016). Diagnosis of Neurodegenerative Diseases: The Clinical Approach. Curr. Alzheimer Res..

[5] Yang J.-H., Li J.-H., Jiang S., Zhou H., Qu L.-H. (2012). ChIPBase: A database for decoding the transcriptional regulation of long non-coding RNA and microRNA genes from ChIP-Seq data. Nucleic Acids Res..

[6] Dahariya S., Paddibhatla I., Kumar S., Raghuwanshi S., Pallepati A., Gutti R.K. (2019). Long non-coding RNA: Classification, biogenesis and functions in blood cells. Mol. Immunol..

[7] Kumar S., Vijayan M., Bhatti J.S., Reddy P. (2017). MicroRNAs as Peripheral Biomarkers in Aging and Age-Related Diseases. Prog. Mol. Biol. Transl. Sci..

[8] Ehu Y.-B., Eli C.-B., Esong N., Ezou Y., Echen S.-D., Eren R.-J., Ewang G. (2016). Diagnostic Value of microRNA for Alzheimer’s Disease: A Systematic Review and Meta-Analysis. Front. Aging Neurosci..

[9] Wu P., Zuo X., Deng H., Liu X., Liu L., Ji A. (2013). Roles of long noncoding RNAs in brain development, functional diversification and neurodegenerative diseases. Brain Res. Bull..

[10] Hubčík L., Galliková D., Pullmannová1 P., Lacinová L., Sulová Z., Hanulová M., Funari S.S., Devínsky F., Uhríková D. (2018). DNA–DOPE–gemini surfactants complexes at low surface charge density: From structure to transfection efficiency. Gen. Physiol. Biophys..

[11] Pritchard C.C., Cheng H.H., Tewari M. (2012). MicroRNA profiling: Approaches and considerations. Nat. Rev. Genet..

[12] Quinlan S., Kenny A., Medina M., Engel T., Jimenez-Mateos E.M. (2017). MicroRNAs in Neurodegenerative Diseases. Int. Rev. Cell Mol. Biol..

[13] Riva P., Ratti A., Venturin M. (2016). The Long Non-Coding RNAs in Neurodegenerative Diseases: Novel Mechanisms of Pathogen-esis. Curr. Alzheimer Res..

[14] Ludwig N., Fehlmann T., Kern F., Gogol M., Maetzler W., Deutscher S., Gurlit S., Schulte C., von Thaler A.-K., Deuschle C. (2019). Machine Learning to Detect Alzheimer’s Disease from Circulating Non-coding RNAs. Genom. Proteom. Bioinform..

[15] Sonntag K.-C. (2010). MicroRNAs and deregulated gene expression networks in neurodegeneration. Brain Res..

[16] Eacker S.M., Dawson T.M., Dawson V. (2009). Understanding microRNAs in neurodegeneration. Nat. Rev. Neurosci..

[17] Hooten N.N., Fitzpatrick M., Wood W.H., De S., Ejiogu N., Zhang Y., Mattison J.A., Becker K., Zonderman A.B., Evans M. (2013). Age-related changes in microRNA levels in serum. Aging.

[18] Cortez M.A., Bueso-Ramos C., Ferdin J., Lopez-Berestein G., Sood A.K., Calin G.A. (2011). MicroRNAs in body fluids—the mix of hormones and biomarkers. Nat. Rev. Clin. Oncol..

[19] Machida T., Tomofuji T., Ekuni D., Maruyama T., Yoneda T., Kawabata Y., Mizuno H., Miyai H., Kunitomo M., Morita M. (2015). MicroRNAs in Salivary Exosome as Potential Biomarkers of Aging. Int. J. Mol. Sci..

[20] El Naqa I., Murphy M.J. (2015). What Is Machine Learning?. Machine Learning in Radiation Oncology.

[21] Deo R.C. (2015). Machine Learning in Medicine. Circulation.

[22] Esteva A., Kuprel B., Novoa R.A., Ko J., Swetter S.M., Blau H.M., Thrun S. (2017). Dermatologist-level classification of skin cancer with deep neural networks. Nature.

[23] Liu J., Wang X., Cheng Y., Zhang L. (2017). Tumor gene expression data classification via sample expansion-based deep learning. Oncotarget.

[24] Rahimy E. (2018). Deep learning applications in ophthalmology. Curr. Opin. Ophthalmol..

[25] Huang C., Mezencev R., McDonald J.F., Vannberg F. (2017). Open source machine-learning algorithms for the prediction of optimal cancer drug therapies. PLoS ONE.

[26] Feinbaum R., Ambros V., Lee R. (2004). The C. elegans Heterochronic Gene lin-4 Encodes Small RNAs with Antisense Complemen-tarity to lin-4. Cell.

[27] O’Brien J., Hayder H., Zayed Y., Peng C. (2018). Overview of MicroRNA Biogenesis, Mechanisms of Actions, and Circulation. Front. Endocrinol..

[28] Mattick J.S. (2018). The State of Long Non-Coding RNA Biology. Non-Coding RNA.

[29] Reinhart B.J., Slack F.J., Basson M., Pasquinelli A.E., Bettinger J.C., Rougvie A.E., Horvitz H.R., Ruvkun G. (2000). The 21-nucleotide let-7 RNA regulates developmental timing in Caenorhabditis elegans. Nature.

[30] Almeida M.I., Reis R., Calin G. (2011). MicroRNA history: Discovery, recent applications, and next frontiers. Mutat. Res. Mol. Mech. Mutagen..

[31] Pasquinelli A.E., Reinhart B.J., Slack F., Martindale M.Q., Kuroda M.I., Maller B., Hayward D.C., Ball E.E., Degnan B., Müller P. (2000). Conservation of the sequence and temporal expression of let-7 heterochronic regulatory RNA. Nature.

[32] Su J.-L., Chen P.-S., Johansson G., Kuo M.-L. (2012). Function and Regulation of Let-7 Family microRNAs. MicroRNA.

[33] Wahid F., Shehzad A., Khan T., Kim Y.Y. (2010). MicroRNAs: Synthesis, mechanism, function, and recent clinical trials. Biochim. Biophys. Acta (BBA) Bioenerg..

[34] Esteller M. (2011). Non-coding RNAs in human disease. Nat. Rev. Genet..

[35] Johnson R., Noble W., Tartaglia G.G., Buckley N.J. (2012). Neurodegeneration as an RNA disorder. Prog. Neurobiol..

[36] Lin J., Teo S., Lam D.H., Jeyaseelan K., Wang S. (2012). MicroRNA-10b pleiotropically regulates invasion, angiogenicity and apoptosis of tumor cells resembling mesenchymal subtype of glioblastoma multiforme. Cell Death Dis..

[37] Heravi-Moussavi A., Anglesio M.S., Cheng S.-W.G., Senz J., Yang W., Prentice L., Fejes A.P., Chow C., Tone A., Kalloger S.E. (2012). Recurrent SomaticDICER1Mutations in Nonepithelial Ovarian Cancers. N. Engl. J. Med..

[38] Rakheja D., Chen K., Liu Y., Shukla A.A., Schmid V., Chang T.-C., Khokhar S., Wickiser J.E., Karandikar N., Malter J.S. (2014). Somatic mutations in DROSHA and DICER1 impair microRNA biogenesis through distinct mechanisms in Wilms tumours. Nat. Commun..

[39] Ha M., Kim V.N. (2014). Regulation of microRNA biogenesis. Nat. Rev. Mol. Cell Biol..

[40] Winter J., Jung S., Keller S., Gregory R.I., Diederichs S. (2009). Many roads to maturity: MicroRNA biogenesis pathways and their regulation. Nat. Cell Biol..

[41] Kim V.N. (2005). MicroRNA biogenesis: Coordinated cropping and dicing. Nat. Rev. Mol. Cell Biol..

[42] Guo L., Lu Z. (2010). The Fate of miRNA* Strand through Evolutionary Analysis: Implication for Degradation as Merely Carrier Strand or Potential Regulatory Molecule?. PLoS ONE.

[43] He B., Huang X., Ma M., Chang Q., Tu Y., Li Q., Zhang K., Hong Y. (2018). Analysis of flash flood disaster characteristics in China from 2011 to 2015. Nat. Hazards.

[44] Wu H., Yang L., Chen L.-L. (2017). The Diversity of Long Noncoding RNAs and Their Generation. Trends Genet..

[45] Brannan C.I., Dees E.C., Ingram R.S., Tilghman S.M. (1990). The product of the H19 gene may function as an RNA. Mol. Cell. Biol..

[46] Lyon M.F. (1961). Gene Action in the X-chromosome of the Mouse (*Mus musculus* L.). Nat. Cell Biol..

[47] Venter J.C., Adams M.D., Myers E.W., Li P.W., Mural R.J., Sutton G.G., Smith H.O., Yandell M., Evans C.A., Holt R.A. (2001). The Sequence of the Human Genome. Science.

[48] Jathar S., Kumar V., Srivastava J., Tripathi V. (2017). Technological Developments in lncRNA Biology. Adv. Exp. Med. Biol..

[49] Gan L., Xu M., Zhang Y., Zhang X., Guo W. (2014). Focusing on long noncoding RNA dysregulation in gastric cancer. Tumor Biol..

[50] Uchida S., Dimmeler S. (2015). Long Noncoding RNAs in Cardiovascular Diseases. Circ. Res..

[51] Wang K.C., Chang H.Y. (2011). Molecular Mechanisms of Long Noncoding RNAs. Mol. Cell.

[52] Hung T., Wang Y., Lin M.F., Koegel A.K., Kotake Y., Grant G.D., Horlings H.M., Shah N., Umbricht C., Wang P. (2011). Extensive and coordinated transcription of noncoding RNAs within cell-cycle promoters. Nat. Genet..

[53] Rinn J.L., Chang H.Y. (2012). Genome Regulation by Long Noncoding RNAs. Annu. Rev. Biochem..

[54] Franco-Zorrilla J.M., Valli A., Todesco M., Mateos I., Puga M.I., Somoza I.R., Leyva A., Weigel D., Garcia J.A., Paz-Ares J. (2007). Target mimicry provides a new mechanism for regulation of microRNA activity. Nat. Genet..

[55] Cao B., Wang T., Qu Q., Kang T., Yang Q. (2018). Long Noncoding RNA SNHG1 Promotes Neuroinflammation in Parkinson’s Disease via Regulating miR-7/NLRP3 Pathway. Neuroscience.

[56] Gupta R.A., Shah N., Wang K.C., Kim J., Horlings H.M., Wong D.J., Tsai M.-C., Hung T., Argani P., Rinn J.L. (2010). Long non-coding RNA HOTAIR reprograms chromatin state to promote cancer metastasis. Nature.

[57] Tsai M.-C., Manor O., Wan Y., Mosammaparast N., Wang J.K., Lan F., Shi Y., Segal E., Chang H.Y. (2010). Long Noncoding RNA as Modular Scaffold of Histone Modification Complexes. Science.

[58] Hanly D.J., Esteller M., Berdasco M. (2018). Interplay between long non-coding RNAs and epigenetic machinery: Emerging targets in cancer?. Philos. Trans. R. Soc. Lond. B Biol. Sci..

[59] Kotake Y., Nakagawa T., Kitagawa K., Suzuki S., Liu N., Kitagawa M., Xiong Y. (2010). Long non-coding RNA ANRIL is required for the PRC2 recruitment to and silencing of p15INK4B tumor suppressor gene. Oncogene.

[60] Di Ruscio A., Ebralidze A.K., Benoukraf T., Amabile G., Goff L., Terragni J., Figueroa M.E., Pontes L.L.D.F., Jorda M.A., Zhang P. (2013). DNMT1-interacting RNAs block gene-specific DNA methylation. Nat. Cell Biol..

[61] Zhang C., Ge S., Gong W., Xu J., Guo Z., Liu Z., Gao X., Wei X., Ge S. (2020). LncRNA ANRIL acts as a modular scaffold of WDR5 and HDAC3 complexes and promotes alteration of the vascular smooth muscle cell phenotype. Cell Death Dis..

[62] Cai X., Cullen B.R. (2007). The imprinted H19 noncoding RNA is a primary microRNA precursor. RNA.

[63] Schmitz S.U., Grote P., Herrmann B.G. (2016). Mechanisms of long noncoding RNA function in development and disease. Cell. Mol. Life Sci..

[64] Wang Y., Pang W.-J., Wei N., Xiong Y., Wu W.-J., Zhao C.-Z., Shen Q.-W., Yang G.-S. (2014). Identification, stability and expression of Sirt1 antisense long non-coding RNA. Gene.

[65] Jacob R., Zander S., Gutschner T. (2017). The Dark Side of the Epitranscriptome: Chemical Modifications in Long Non-Coding RNAs. Int. J. Mol. Sci..

[66] Liu N.-K., Xu X.-M. (2011). MicroRNA in central nervous system trauma and degenerative disorders. Physiol. Genom..

[67] Clark B.S., Blackshaw S. (2017). Understanding the Role of lncRNAs in Nervous System Development. Adv. Exp. Med. Biol..

[68] Ng S.-Y., Johnson R., Stanton L.W. (2011). Human long non-coding RNAs promote pluripotency and neuronal differentiation by association with chromatin modifiers and transcription factors. EMBO J..

[69] Ma Q., Zhang L., Pearce W. (2019). MicroRNAs in brain development and cerebrovascular pathophysiology. Am. J. Physiol. Physiol..

[70] Ramos A.D., Andersen R., Liu S.J., Nowakowski T.J., Hong S.J., Gertz C.C., Salinas R.D., Zarabi H., Kriegstein A.R., Lim D.A. (2015). The Long Noncoding RNA Pnky Regulates Neuronal Differentiation of Embryonic and Postnatal Neural Stem Cells. Cell Stem Cell.

[71] Ponjavic J., Oliver P.L., Lunter G., Ponting C.P. (2009). Genomic and Transcriptional Co-Localization of Protein-Coding and Long Non-Coding RNA Pairs in the Developing Brain. PLoS Genet..

[72] Mercer T.R., Dinger M.E., Sunkin S.M., Mehler M.F., Mattick J.S. (2008). Specific expression of long noncoding RNAs in the mouse brain. Proc. Natl. Acad. Sci. USA.

[73] Lin M., Pedrosa E., Shah A., Hrabovsky A., Maqbool S., Zheng D., Lachman H.M. (2011). RNA-Seq of Human Neurons Derived from iPS Cells Reveals Candidate Long Non-Coding RNAs Involved in Neurogenesis and Neuropsychiatric Disorders. PLoS ONE.

[74] Lipovich L., Tarca A.L., Cai J., Jia H., Chugani H.T., Sterner K., Grossman L.I., Uddin M., Hof P.R., Sherwood C.C. (2013). Developmental Changes in the Transcriptome of Human Cerebral Cortex Tissue: Long Noncoding RNA Transcripts. Cereb. Cortex.

[75] Muslimov I.A., Banker G., Brosius J., Tiedge H. (1998). Activity-dependent Regulation of Dendritic BC1 RNA in Hippocampal Neurons in Culture. J. Cell Biol..

[76] Tripathi V., Ellis J.D., Shen Z., Song D.Y., Pan Q., Watt A.T., Freier S.M., Bennett C.F., Sharma A., Bubulya P.A. (2010). The Nuclear-Retained Noncoding RNA MALAT1 Regulates Alternative Splicing by Modulating SR Splicing Factor Phosphorylation. Mol. Cell.

[77] Zhong J., Chuang S.-C., Bianchi R., Zhao W., Lee H., Fenton A., Wong R.K.S., Tiedge H. (2009). BC1 Regulation of Metabotropic Glutamate Receptor-Mediated Neuronal Excitability. J. Neurosci..

[78] Bernard D., Prasanth K.V., Tripathi V., Colasse S., Nakamura T., Xuan Z., Zhang M.Q., Sedel F., Jourdren L., Coulpier F. (2010). A long nuclear-retained non-coding RNA regulates synaptogenesis by modulating gene expression. EMBO J..

[79] Smirnova L., Gräfe A., Seiler A., Schumacher S., Nitsch R., Wulczyn F.G. (2005). Regulation of miRNA expression during neural cell specification. Eur. J. Neurosci..

[80] Meza-Sosa K.F., Epedraza-Alva G., Pérez-Martínez L. (2014). microRNAs: Key triggers of neuronal cell fate. Front. Cell. Neurosci..

[81] Szulwach K.E., Li X., Smrt R.D., Li Y., Luo Y., Lin L., Santistevan N., Li W., Zhao X., Jin P. (2010). Cross talk between microRNA and epigenetic regulation in adult neurogenesis. J. Cell Biol..

[82] Hu Z., Zhao J., Hu T., Luo Y., Zhu J., Li Z. (2015). miR-501-3p mediates the activity-dependent regulation of the expression of AMPA receptor subunit GluA1. J. Cell Biol..

[83] Mohammed C.P.D., Rhee H., Phee B., Kim K., Kim H., Lee H., Park J.H., Jung J.H., Kim J.Y., Kim H. (2016). miR-204 downregulates EphB2 in aging mouse hippocampal neurons. Aging Cell.

[84] Lukiw W.J., Dua P., Pogue A.I., Eicken C., Hill J.M. (2011). Upregulation of Micro RNA-146a (miRNA-146a), A Marker for Inflammatory Neurodegeneration, in Sporadic Creutzfeldt–Jakob Disease (sCJD) and Gerstmann–Straussler–Scheinker (GSS) Syndrome. J. Toxicol. Environ. Health Part A.

[85] Iyer A., Zurolo E., Prabowo A., Fluiter K., Spliet W.G.M., Van Rijen P.C., Gorter J.A., Aronica E. (2012). MicroRNA-146a: A Key Regulator of Astrocyte-Mediated Inflammatory Response. PLoS ONE.

[86] Harraz M.M., Eacker S.M., Wang X., Dawson T.M., Dawson V.L. (2012). MicroRNA-223 is neuroprotective by targeting glutamate receptors. Proc. Natl. Acad. Sci. USA.

[87] Eacker S.M., Dawson T.M., Dawson V.L. (2013). The interplay of microRNA and neuronal activity in health and disease. Front. Cell. Neurosci..

[88] Krol J., Busskamp V., Markiewicz I., Stadler M.B., Ribi S., Richter J., Duebel J., Bicker S., Fehling H.J., Schübeler D. (2010). Characterizing Light-Regulated Retinal MicroRNAs Reveals Rapid Turnover as a Common Property of Neuronal MicroRNAs. Cell.

[89] Sierksma A., Lu A., Salta E., Eynden E.V., Callaerts-Vegh Z., D’Hooge R., Blum D., Buée L., Fiers M., De Strooper B. (2018). Deregulation of neuronal miRNAs induced by amyloid-β or TAU pathology. Mol. Neurodegener..

[90] Massone S., Vassallo I., Fiorino G., Castelnuovo M., Barbieri F., Borghi R., Tabaton M., Robello M., Gatta E., Russo C. (2011). 17A, a novel non-coding RNA, regulates GABA B alternative splicing and signaling in response to inflammatory stimuli and in Alzheimer disease. Neurobiol. Dis..

[91] Ke S., Yang Z., Yang F., Wang X., Tan J., Liao B. (2019). Long Noncoding RNA NEAT1 Aggravates Aβ-Induced Neuronal Damage by Targeting miR-107 in Alzheimer’s Disease. Yonsei Med. J..

[92] Wang S., Zhang X., Guo Y., Rong H., Liu T. (2017). The long noncoding RNA HOTAIR promotes Parkinson’s disease by upregulating LRRK2 expression. Oncotarget.

[93] Caggiu E., Paulus K., Mameli G., Arru G., Sechi G.P., Sechi L.A. (2018). Differential expression of miRNA 155 and miRNA 146a in Parkinson’s disease patients. eNeurologicalSci.

[94] Swarbrick S., Wragg N., Ghosh S., Stolzing A. (2019). Systematic Review of miRNA as Biomarkers in Alzheimer’s Disease. Mol. Neurobiol..

[95] Dugger B.N., Dickson D.W. (2017). Pathology of Neurodegenerative Diseases. Cold Spring Harb. Perspect. Biol..

[96] Amor S., Puentes F., Baker D., Van Der Valk P. (2010). Inflammation in neurodegenerative diseases. Immunology.

[97] Mazon J.N., de Mello A.H., Ferreira G.K., Rezin G. (2017). The impact of obesity on neurodegenerative diseases. Life Sci..

[98] De Guire V., Robitaille R., Tétreault N., Guérin R., Ménard C., Bambace N., Sapieha P. (2013). Circulating miRNAs as sensitive and specific biomarkers for the diagnosis and monitoring of human diseases: Promises and challenges. Clin. Biochem..

[99] Chen X., Liang H., Zhang J., Zen K., Zhang C.-Y. (2012). Secreted microRNAs: A new form of intercellular communication. Trends Cell Biol..

[100] Durães F., Pinto M., Sousa M.E. (2018). Old Drugs as New Treatments for Neurodegenerative Diseases. Pharmaceuticals.

[101] Sudhakar V., Richardson R.M. (2019). Gene Therapy for Neurodegenerative Diseases. Neurotherapeutics.

[102] Maciotta S., Emeregalli M., Etorrente Y. (2013). The involvement of microRNAs in neurodegenerative diseases. Front. Cell. Neurosci..

[103] Weller J., Budson A. (2018). Current understanding of Alzheimer’s disease diagnosis and treatment. F1000Research.

[104] Reddy P.H. (2011). Abnormal tau, mitochondrial dysfunction, impaired axonal transport of mitochondria, and synaptic deprivation in Alzheimer’s disease. Brain Res..

[105] Gao Y.-L., Wang N., Sun F.-R., Cao X.-P., Zhang W., Yu J.-T. (2018). Tau in neurodegenerative disease. Ann. Transl. Med..

[106] (2016). Alzheimer’s Association 2016 Alzheimer’s disease facts and figures. Alzheimer’s Dement..

[107] Selkoe D.J. (2001). Alzheimer’s Disease: Genes, Proteins, and Therapy. Physiol. Rev..

[108] Galimberti D., Villa C., Fenoglio C., Serpente M., Ghezzi L., Cioffi S.M., Arighi A., Fumagalli G.G., Scarpini E. (2014). Circulating miRNAs as Potential Biomarkers in Alzheimer’s Disease. J. Alzheimer’s Dis..

[109] Ebhatnagar S., Echertkow H., Schipper H.M., Eyuan Z., Eshetty V., Ejenkins S., Ejones T., Ewang E. (2014). Increased microRNA-34c abundance in Alzheimer’s disease circulating blood plasma. Front. Mol. Neurosci..

[110] Zovoilis A., Agbemenyah H.Y., Agís-Balboa R.C., Stilling R., Edbauer D., Rao P., Farinelli L., Delalle I., Schmitt A., Falkai P. (2011). microRNA-34c is a novel target to treat dementias. EMBO J..

[111] Faghihi M.A., Modarresi F., Khalil A.M., Wood D.E., Sahagan B.G., Morgan T.E., Finch C.E., Iii G.S.L., Kenny P.J., Wahlestedt C. (2008). Expression of a noncoding RNA is elevated in Alzheimer’s disease and drives rapid feed-forward regulation of β-secretase. Nat. Med..

[112] Laird F.M., Cai H., Savonenko A.V., Farah M.H., He K., Melnikova T., Wen H., Chiang H.-C., Xu G., Koliatsos V.E. (2005). BACE1, a Major Determinant of Selective Vulnerability of the Brain to Amyloid- Amyloidogenesis, is Essential for Cognitive, Emotional, and Synaptic Functions. J. Neurosci..

[113] Magistri M., Velmeshev D., Makhmutova M., Faghihi M.A. (2015). Transcriptomics Profiling of Alzheimer’s Disease Reveal Neurovascular Defects, Altered Amyloid-β Homeostasis, and Deregulated Expression of Long Noncoding RNAs. J. Alzheimer’s Dis..

[114] Zhao L.Y., Niu Y., Santiago A., Liu J., Albert S.H., Robertson K., Liao D. (2006). An EBF3-Mediated Transcriptional Program That Induces Cell Cycle Arrest and Apoptosis. Cancer Res..

[115] Satoh J.-I., Kino Y., Niida S. (2015). MicroRNA-Seq Data Analysis Pipeline to Identify Blood Biomarkers for Alzheimer’s Disease from Public Data. Biomark. Insights.

[116] Fransquet P., Ryan J. (2018). Micro RNA as a potential blood-based epigenetic biomarker for Alzheimer’s disease. Clin. Biochem..

[117] Zhang W., Zhao H., Wu Q., Xu W., Xia M. (2018). Knockdown of BACE1-AS by siRNA improves memory and learning behaviors in Alzheimer’s disease animal model. Exp. Ther. Med..

[118] Schipper H.M., Maes O.C., Chertkow H.M., Wang E. (2007). MicroRNA Expression in Alzheimer Blood Mononuclear Cells. Gene Regul. Syst. Biol..

[119] Tan L., Yu J.-T., Liu Q.-Y., Tan M.-S., Zhang W., Hu N., Wang Y.-L., Sun L., Jiang T., Tan L. (2014). Circulating miR-125b as a biomarker of Alzheimer’s disease. J. Neurol. Sci..

[120] Tan L., Yu J.-T., Tan M.-S., Liu Q.-Y., Wang H.-F., Zhang W., Jiang T., Tan L. (2014). Genome-Wide Serum microRNA Expression Profiling Identifies Serum Biomarkers for Alzheimer’s Disease. J. Alzheimer’s Dis..

[121] Cheng L., Doecke J.D., Sharples R.A., Villemagne V.L., Fowler C.J., Rembach A., Martins R., Rowe C.C., Macaulay S.L., Masters C. (2015). Prognostic serum miRNA biomarkers associated with Alzheimer’s disease shows concordance with neuropsychological and neuroimaging assessment. Mol. Psychiatry.

[122] Kumar P., Dezso Z., MacKenzie C., Oestreicher J., Agoulnik S., Byrne M., Bernier F., Yanagimachi M., Aoshima K., Oda Y. (2013). Circulating miRNA Biomarkers for Alzheimer’s Disease. PLoS ONE.

[123] Lugli G., Cohen A.M., Bennett D.A., Shah R., Fields C., Hernandez A.G., Smalheiser N.R. (2015). Plasma Exosomal miRNAs in Persons with and without Alzheimer Disease: Altered Expression and Prospects for Biomarkers. PLoS ONE.

[124] Denk J., Boelmans K., Siegismund C.S., Lassner D., Arlt S., Jahn H. (2015). MicroRNA Profiling of CSF Reveals Potential Biomarkers to Detect Alzheimer‘s Disease. PLoS ONE.

[125] Cogswell J.P., Ward J., Taylor I.A., Waters M., Shi Y., Cannon B., Kelnar K., Kemppainen J., Brown D., Chen C. (2008). Identification of miRNA Changes in Alzheimer’s Disease Brain and CSF Yields Putative Biomarkers and Insights into Disease Pathways. J. Alzheimer’s Dis..

[126] Alexandrov P.N., Dua P., Hill J.M., Bhattacharjee S., Zhao Y., Lukiw W.J. (2012). microRNA (miRNA) speciation in Alzheimer’s disease (AD) cerebrospinal fluid (CSF) and extracellular fluid (ECF). Int. J. Biochem. Mol. Boil..

[127] Lukiw W.J. (2007). Micro-RNA speciation in fetal, adult and Alzheimer’s disease hippocampus. NeuroReport.

[128] Yao J., Wang X., Li Y., Shan K., Yang H., Wang Y., Yao M., Liu C., Li X., Shen Y. (2016). Long non-coding RNA MALAT 1 regulates retinal neurodegeneration through CREB signaling. EMBO Mol. Med..

[129] Geekiyanage H., Chan C. (2011). MicroRNA-137/181c Regulates Serine Palmitoyltransferase and In Turn Amyloid, Novel Targets in Sporadic Alzheimer’s Disease. J. Neurosci..

[130] Jayadev S., Case A., Alajajian B., Eastman A.J., Möller T., Garden G.A. (2013). Presenilin 2 influences miR146 level and activity in microglia. J. Neurochem..

[131] Hebert S., De Strooper B. (2009). Alterations of the microRNA network cause neurodegenerative disease. Trends Neurosci..

[132] Fang M., Wang J., Zhang X., Geng Y., Hu Z., Rudd J.A., Ling S., Chen W., Han S. (2012). The miR-124 regulates the expression of BACE1/β-secretase correlated with cell death in Alzheimer’s disease. Toxicol. Lett..

[133] Dickson J.R., Kruse C., Montagna D.R., Finsen B., Wolfe M.S. (2013). Alternative polyadenylation and miR-34 family members regulate tau expression. J. Neurochem..

[134] Absalon S., Kochanek D.M., Raghavan V., Krichevsky A.M. (2013). MiR-26b, Upregulated in Alzheimer’s Disease, Activates Cell Cycle Entry, Tau-Phosphorylation, and Apoptosis in Postmitotic Neurons. J. Neurosci..

[135] Patel N., Hoang D., Miller N., Ansaloni S., Huang Q., Rogers J.T., Lee J.C., Saunders A.J. (2008). MicroRNAs can regulate human APP levels. Mol. Neurodegener..

[136] Long J., Lahiri D.K. (2011). MicroRNA-101 downregulates Alzheimer’s amyloid-β precursor protein levels in human cell cultures and is differentially expressed. Biochem. Biophys. Res. Commun..

[137] Smith P., Al Hashimi A., Girard J., DeLay C., Hebert S. (2010). In vivo regulation of amyloid precursor protein neuronal splicing by microRNAs. J. Neurochem..

[138] Hébert S., Horré K., Nicolaï L., Papadopoulou A.S., Mandemakers W., Silahtaroglu A., Kauppinen S., Delacourte A., De Strooper B. (2008). Loss of microRNA cluster miR-29a/b-1 in sporadic Alzheimer’s disease correlates with increased BACE1/β-secretase expression. Proc. Natl. Acad. Sci. USA.

[139] Hu Y.-K., Wang X., Li L., Du Y.-H., Ye H.-T., Li C.-Y. (2013). MicroRNA-98 induces an Alzheimer’s disease-like disturbance by targeting insulin-like growth factor. Neurosci. Bull..

[140] Cao M., Li H., Zhao J., Cui J., Hu G. (2019). Identification of age- and gender-associated long noncoding RNAs in the human brain with Alzheimer’s disease. Neurobiol. Aging.

[141] Spreafico M., Grillo B., Rusconi F., Battaglioli E., Venturin M. (2018). Multiple Layers of CDK5R1 Regulation in Alzheimer’s Disease Implicate Long Non-Coding RNAs. Int. J. Mol. Sci..

[142] Luo Q., Chen Y. (2016). Long noncoding RNAs and Alzheimer’s disease. Clin. Interv. Aging.

[143] Zhou M., Zhao H., Wang X., Sun J., Su J. (2019). Analysis of long noncoding RNAs highlights region-specific altered expression patterns and diagnostic roles in Alzheimer’s disease. Briefings Bioinform..

[144] Zhou X., Xu J. (2015). Identification of Alzheimer’s disease–associated long noncoding RNAs. Neurobiol. Aging.

[145] Tysnes O.-B., Storstein A. (2017). Epidemiology of Parkinson’s disease. J. Neural Transm..

[146] Beitz J.M. (2014). Parkinson s disease: A review. Front. Biosci..

[147] Armstrong M.J., Okun M.S. (2020). Diagnosis and Treatment of Parkinson Disease. JAMA.

[148] Poewe W., Seppi K., Tanner C.M., Halliday G.M., Brundin P., Volkmann J., Schrag A.E., Lang A.E. (2017). Parkinson disease. Nat. Rev. Dis. Prim..

[149] Kim J., Inoue K., Ishii J., Vanti W., Voronov S.V., Murchison E., Hannon G., Abeliovich A. (2007). A MicroRNA Feedback Circuit in Midbrain Dopamine Neurons. Science.

[150] Elkouris M., Kouroupi G., Vourvoukelis A., Papagiannakis N., Kaltezioti V., Matsas R., Stefanis L., Xilouri M., Politis P.K. (2019). Long Non-coding RNAs Associated With Neurodegeneration-Linked Genes Are Reduced in Parkinson’s Disease Patients. Front. Cell. Neurosci..

[151] Wang G., van der Walt J.M., Mayhew G., Li Y.-J., Züchner S., Scott W.K., Martin E.R., Vance J.M. (2008). Variation in the miRNA-433 Binding Site of FGF20 Confers Risk for Parkinson Disease by Overexpression of α-Synuclein. Am. J. Hum. Genet..

[152] Mahul-Mellier A.-L., Burtscher J., Maharjan N., Weerens L., Croisier M., Kuttler F., Leleu M., Knott G.W., Lashuel H.A. (2020). The process of Lewy body formation, rather than simply α-synuclein fibrillization, is one of the major drivers of neurodegeneration. Proc. Natl. Acad. Sci. USA.

[153] Junn E., Lee K.-W., Jeong B.S., Chan T.W., Im J.-Y., Mouradian M.M. (2009). Repression of α-synuclein expression and toxicity by microRNA-7. Proc. Natl. Acad. Sci. USA.

[154] Zhang Q.-S., Wang Z.-H., Zhang J.-L., Duan Y.-L., Li G.-F., Zheng D.-L. (2016). Beta-asarone protects against MPTP-induced Parkinson’s disease via regulating long non-coding RNA MALAT1 and inhibiting α-synuclein protein expression. Biomed. Pharmacother..

[155] Margis R., Margis R., Rieder C.R. (2011). Identification of blood microRNAs associated to Parkinsońs disease. J. Biotechnol..

[156] Dos Santos M.C.T., Bell R., Da Costa A.N. (2016). Recent developments in circulating biomarkers in Parkinson’s disease: The potential use of miRNAs in a clinical setting. Bioanalysis.

[157] Chi L.-M., Wang L.-P., Jiao D. (2019). Identification of Differentially Expressed Genes and Long Noncoding RNAs Associated with Parkinson’s Disease. Park. Dis..

[158] Fan Y., Li J., Yang Q., Gong C., Gao H., Mao Z., Yuan X., Zhu S., Xue Z. (2019). Dysregulated Long Non-coding RNAs in Parkinson’s Disease Contribute to the Apoptosis of Human Neuroblastoma Cells. Front. Neurosci..

[159] Zhou Y., Gu C., Li J., Zhu L., Huang G., Dai J., Huang H. (2018). Aberrantly expressed long noncoding RNAs and genes in Parkinson’s disease. Neuropsychiatr. Dis. Treat..

[160] Cardo L.F., Coto E., de Mena L., Ribacoba R., Moris G., Menéndez M., Álvarez L.D.M. (2013). Profile of microRNAs in the plasma of Parkinson’s disease patients and healthy controls. J. Neurol..

[161] Dong H., Wang C., Lu S., Yu C., Huang L., Feng W., Xu H., Chen X., Zen K., Yan Q. (2015). A panel of four decreased serum microRNAs as a novel biomarker for early Parkinson’s disease. Biomarkers.

[162] Vallelunga A., Ragusa M., Di Mauro S., Iannitti T., Pilleri M., Biundo R., Weis L., Di Pietro C.S., De Iuliis A., Nicoletti A. (2014). Identification of circulating microRNAs for the differential diagnosis of Parkinson’s disease and Multiple System Atrophy. Front. Cell. Neurosci..

[163] Botta-Orfila T., Morató X., Compta Y., Lozano J.J., Falgàs N., Valldeoriola F., Pont-Sunyer C., Vilas D., Mengual L., Fernández M. (2014). Identification of blood serum micro-RNAs associated with idiopathic andLRRK2Parkinson’s disease. J. Neurosci. Res..

[164] Doxakis E. (2010). Post-transcriptional Regulation of α-Synuclein Expression by mir-7 and mir-153. J. Biol. Chem..

[165] Cho H.J., Liu G., Jin S.M., Parisiadou L., Xie C., Yu J., Sun L., Ma B., Ding J., Vancraenenbroeck R. (2013). MicroRNA-205 regulates the expression of Parkinson’s disease-related leucine-rich repeat kinase 2 protein. Hum. Mol. Genet..

[166] Gehrke S., Imai Y., Sokol N., Lu B. (2010). Pathogenic LRRK2 negatively regulates microRNA-mediated translational repression. Nat. Cell Biol..

[167] Wang J., Zhang P.-C., Lu H.-F., Ma N., Wang S., Mao H.-Q., Leong K.W. (2002). New polyphosphoramidate with a spermidine side chain as a gene carrier. J. Control. Release.

[168] Davis E., Caiment F., Tordoir X., Cavaillé J., Ferguson-Smith A., Cockett N., Georges M., Charlier C. (2005). RNAi-Mediated Allelic trans-Interaction at the Imprinted Rtl1/Peg11 Locus. Curr. Biol..

[169] Wei C.-W., Luo T., Zou S.-S., Wu A.-S. (2018). The Role of Long Noncoding RNAs in Central Nervous System and Neurodegenerative Diseases. Front. Behav. Neurosci..

[170] Cattaneo E., Zuccato C., Tartari M. (2005). Normal huntingtin function: An alternative approach to Huntington’s disease. Nat. Rev. Neurosci..

[171] Zuccato C., Valenza M., Cattaneo E. (2010). Molecular Mechanisms and Potential Therapeutical Targets in Huntington’s Disease. Physiol. Rev..

[172] Ghosh R., Tabrizi S.J., Nóbrega C., Pereira de Almeida L. (2018). Clinical Features of Huntington’s Disease. Polyglutamine Disorders. Advances in Experimental Medicine and Biology.

[173] Snowden J.S. (2017). The Neuropsychology of Huntington’s Disease. Arch. Clin. Neuropsychol..

[174] McColgan P., Tabrizi S.J. (2018). Huntington’s disease: A clinical review. Eur. J. Neurol..

[175] Wyant K.J., Ridder A.J., Dayalu P. (2017). Huntington’s Disease—Update on Treatments. Curr. Neurol. Neurosci. Rep..

[176] Johnson R., Zuccato C., Belyaev N.D., Guest D., Cattaneo E., Buckley N. (2008). A microRNA-based gene dysregulation pathway in Huntington’s disease. Neurobiol. Dis..

[177] Chen G., Ma Q., Goswami D., Shang J., Miller G.M. (2017). Modulation of nuclear REST by alternative splicing: A potential therapeutic target for Huntington’s disease. J. Cell. Mol. Med..

[178] Baldelli P., Meldolesi J. (2015). The Transcription Repressor REST in Adult Neurons: Physiology, Pathology, and Diseases. Eneuro.

[179] Conaco C., Otto S., Han J.-J., Mandel G. (2006). Reciprocal actions of REST and a microRNA promote neuronal identity. Proc. Natl. Acad. Sci. USA.

[180] Vo N., Klein M.E., Varlamova O., Keller D.M., Yamamoto T., Goodman R.H., Impey S. (2005). From The Cover: A cAMP-response element binding protein-induced microRNA regulates neuronal morphogenesis. Proc. Natl. Acad. Sci. USA.

[181] Packer A.N., Xing Y., Harper S.Q., Jones L., Davidson B.L. (2008). The Bifunctional microRNA miR-9/miR-9* Regulates REST and CoREST and Is Downregulated in Huntington’s Disease. J. Neurosci..

[182] Johnson R., Richter N., Jauch R., Gaughwin P.M., Zuccato C., Cattaneo E., Stanton L.W. (2010). Human accelerated region 1 noncoding RNA is repressed by REST in Huntington’s disease. Physiol. Genom..

[183] Díez-Planelles C., Sánchez-Lozano P., Crespo M.D.C., Gil Zamorano J., Ribacoba R., González N., Suárez E., Martínez-Descals A., Camblor P.M., Álvarez V. (2016). Circulating microRNAs in Huntington’s disease: Emerging mediators in metabolic impairment. Pharmacol. Res..

[184] Martí E., Pantano L., Bañez-Coronel M., Llorens F., Miñones-Moyano E., Porta S., Sumoy L., Ferrer I., Estivill X. (2010). A myriad of miRNA variants in control and Huntington’s disease brain regions detected by massively parallel sequencing. Nucleic Acids Res..

[185] Hu G., Niu F., Humburg B.A., Liao K., Bendi V.S., Callen S., Fox H.S., Buch S. (2018). Molecular mechanisms of long noncoding RNAs and their role in disease pathogenesis. Oncotarget.

[186] Sunwoo J.-S., Lee S.-T., Im W., Lee M., Byun J.-I., Jung K.-H., Park K.-I., Jung K.-Y., Lee S.K., Chu K. (2017). Altered Expression of the Long Noncoding RNA NEAT1 in Huntington’s Disease. Mol. Neurobiol..

[187] Johnson R. (2012). Long non-coding RNAs in Huntington’s disease neurodegeneration. Neurobiol. Dis..

[188] Sparber P., Filatova A., Khantemirova M., Skoblov M. (2019). The role of long non-coding RNAs in the pathogenesis of hereditary diseases. BMC Med. Genom..

[189] Chung D.W., Rudnicki D.D., Yu L., Margolis R.L. (2011). A natural antisense transcript at the Huntington’s disease repeat locus regulates HTT expression. Hum. Mol. Genet..

[190] Toivonen J.M., Manzano R., Oliván S., Zaragoza P., García-Redondo A., Osta R. (2014). MicroRNA-206: A Potential Circulating Biomarker Candidate for Amyotrophic Lateral Sclerosis. PLoS ONE.

[191] Oskarsson B., Gendron T.F., Staff N.P. (2018). Amyotrophic Lateral Sclerosis: An Update for 2018. Mayo Clin. Proc..

[192] Kiernan M.C., Vucic S., Cheah B.C., Turner M., Eisen A., Hardiman O., Burrell J., Zoing M.C. (2011). Amyotrophic lateral sclerosis. Lancet.

[193] Dardiotis E., Aloizou A.-M., Siokas V., Patrinos G.P., Deretzi G., Mitsias P., Aschner M., Tsatsakis A. (2018). The Role of MicroRNAs in Patients with Amyotrophic Lateral Sclerosis. J. Mol. Neurosci..

[194] Waller R., Goodall E., Milo M., Cooper-Knock J., Da Costa M., Hobson E., Kazoka M., Wollff H., Heath P.R., Shaw P. (2017). Serum miRNAs miR-206, 143-3p and 374b-5p as potential biomarkers for amyotrophic lateral sclerosis (ALS). Neurobiol. Aging.

[195] De Andrade H.M., de Albuquerque M., Avansini S., Rocha C.D.S., Dogini D., Nucci A., Carvalho B., Lopes-Cendes I., França M.C. (2016). MicroRNAs-424 and 206 are potential prognostic markers in spinal onset amyotrophic lateral sclerosis. J. Neurol. Sci..

[196] Ma G., Wang Y., Li Y., Cui L., Zhao Y., Zhao B., Li K. (2015). MiR-206, a Key Modulator of Skeletal Muscle Development and Disease. Int. J. Biol. Sci..

[197] Williams A.H., Valdez G., Moresi V., Qi X., McAnally J., Elliott J.L., Bassel-Duby R., Sanes J.R., Olson E.N. (2009). MicroRNA-206 Delays ALS Progression and Promotes Regeneration of Neuromuscular Synapses in Mice. Science.

[198] Haramati S., Chapnik E., Sztainberg Y., Eilam R., Zwang R., Gershoni N., McGlinn E., Heiser P., Wills A.-M., Wirguin I. (2010). miRNA malfunction causes spinal motor neuron disease. Proc. Natl. Acad. Sci. USA.

[199] Shioya M., Obayashi S., Tabunoki H., Arima K., Saito Y., Ishida T., Satoh J. (2010). Aberrant microRNA expression in the brains of neurodegenerative diseases: miR-29a decreased in Alzheimer disease brains targets neurone navigator. Neuropathol. Appl. Neurobiol..

[200] Wakabayashi K., Mori F., Kakita A., Takahashi H., Utsumi J., Sasaki H. (2014). Analysis of microRNA from archived formalin-fixed paraffin-embedded specimens of amyotrophic lateral sclerosis. Acta Neuropathol. Commun..

[201] De Felice B., Annunziata A., Fiorentino G., Borra M., Biffali E., Coppola C., Cotrufo R., Brettschneider J., Giordana M.L., Dalmay T. (2014). miR-338-3p is over-expressed in blood, CFS, serum and spinal cord from sporadic amyotrophic lateral sclerosis patients. Neurogenetics.

[202] Barik S. (2008). An intronic microRNA silences genes that are functionally antagonistic to its host gene. Nucleic Acids Res..

[203] Nishimoto Y., Nakagawa S., Hirose T., Okano H.J., Takao M., Shibata S., Suyama S., Kuwako K.-I., Imai T., Murayama S. (2013). The long non-coding RNA nuclear-enriched abundant transcript 1_2 induces paraspeckle formation in the motor neuron during the early phase of amyotrophic lateral sclerosis. Mol. Brain.

[204] Gagliardi S., Zucca S., Pandini C., Diamanti L., Bordoni M., Sproviero D., Arigoni M., Olivero M., Pansarasa O., Ceroni M. (2018). Long non-coding and coding RNAs characterization in Peripheral Blood Mononuclear Cells and Spinal Cord from Amyotrophic Lateral Sclerosis patients. Sci. Rep..

[205] Gagliardi S., Pandini C., Garofalo M., Bordoni M., Pansarasa O., Cereda C. (2018). Long non coding RNAs and ALS: Still much to do. Non-Coding RNA Res..

[206] Freischmidt A., Müller K., Zondler L., Weydt P., Volk A.E., Božič A.L., Walter M., Bonin M., Mayer B., Von Arnim C.A.F. (2014). Serum microRNAs in patients with genetic amyotrophic lateral sclerosis and pre-manifest mutation carriers. Brain.

[207] Freischmidt A., Müller K., Ludolph A.C., Weishaupt J.H. (2013). Systemic dysregulation of TDP-43 binding microRNAs in amyotrophic lateral sclerosis. Acta Neuropathol. Commun..

[208] Freischmidt A., Müller K., Zondler L., Weydt P., Mayer B., von Arnim C.A., Hübers A., Dorst J., Otto M., Holzmann K. (2015). Serum microRNAs in sporadic amyotrophic lateral sclerosis. Neurobiol. Aging.

[209] Matamala J.M., Arias-Carrasco R., Sanchez C., Uhrig M., Bargsted L., Matus S., Maracaja-Coutinho V., Abarzua S., van Zundert B., Verdugo R. (2018). Genome-wide circulating microRNA expression profiling reveals potential biomarkers for amyotrophic lateral sclerosis. Neurobiol. Aging.

[210] Takahashi I., Hama Y., Matsushima M., Hirotani M., Kano T., Hohzen H., Yabe I., Utsumi J., Sasaki H. (2015). Identification of plasma microRNAs as a biomarker of sporadic Amyotrophic Lateral Sclerosis. Mol. Brain.

[211] Sheinerman K.S., Toledo J., Tsivinsky V.G., Irwin D., Grossman M., Weintraub D., Hurtig H.I., Chen-Plotkin A., Wolk D.A., McCluskey L.F. (2017). Circulating brain-enriched microRNAs as novel biomarkers for detection and differentiation of neurodegenerative diseases. Alzheimer’s Res. Ther..

[212] De Felice B., Guida M., Guida M., Coppola C., De Mieri G., Cotrufo R. (2012). A miRNA signature in leukocytes from sporadic amyotrophic lateral sclerosis. Gene.

[213] Liguori M., Nuzziello N., Introna A., Consiglio A., Licciulli F., D’Errico E., Scarafino A., Distaso E., Simone I.L. (2018). Dysregulation of MicroRNAs and Target Genes Networks in Peripheral Blood of Patients with Sporadic Amyotrophic Lateral Sclerosis. Front. Mol. Neurosci..

[214] Chen Y., Wei Q., Chen X., Li C., Cao B., Ou R., Hadano S., Shang H.-F. (2016). Aberration of miRNAs Expression in Leukocytes from Sporadic Amyotrophic Lateral Sclerosis. Front. Mol. Neurosci..

[215] Benigni M., Ricci C., Jones A.R., Giannini F., Al-Chalabi A., Battistini S. (2016). Identification of miRNAs as Potential Biomarkers in Cerebrospinal Fluid from Amyotrophic Lateral Sclerosis Patients. NeuroMol. Med..

[216] Russell A.P., Wada S., Vergani L., Hock M.B., Lamon S., Léger B., Ushida T., Cartoni R., Wadley G., Hespel P. (2013). Disruption of skeletal muscle mitochondrial network genes and miRNAs in amyotrophic lateral sclerosis. Neurobiol. Dis..

[217] Campos-Melo D., Droppelmann C.A., He Z., Volkening K., Strong M.J. (2013). Altered microRNA expression profile in amyotrophic lateral sclerosis: A role in the regulation of NFL mRNA levels. Mol. Brain.

[218] Koval E.D., Shaner C., Zhang P., Du Maine X., Fischer K., Tay J., Chau B.N., Wu G.F., Miller T.M. (2013). Method for widespread microRNA-155 inhibition prolongs survival in ALS-model mice. Hum. Mol. Genet..

[219] Figueroa-Romero C., Hur J., Lunn J.S., Paez-Colasante X., Bender D.E., Yung R., Sakowski S.A., Feldman E.L. (2016). Expression of microRNAs in human post-mortem amyotrophic lateral sclerosis spinal cords provides insight into disease mechanisms. Mol. Cell. Neurosci..

[220] Ishtiaq M., Campos-Melo D., Volkening K., Strong M.J. (2014). Analysis of Novel NEFL mRNA Targeting microRNAs in Amyotrophic Lateral Sclerosis. PLoS ONE.

[221] Bayat A. (2002). Science, medicine, and the future: Bioinformatics. BMJ.

[222] Chen G., Wang Z., Wang D., Qiu C., Liu M., Chen X., Zhang Q., Yan G., Cui Q. (2012). LncRNADisease: A database for long-non-coding RNA-associated diseases. Nucleic Acids Res..

[223] Bao Z., Yang Z., Huang Z., Zhou Y., Cui Q., Dong D. (2019). LncRNADisease 2.0: An updated database of long non-coding RNA-associated diseases. Nucleic Acids Res..

[224] Zhao H., Shi J., Zhang Y., Xie A., Yu L., Zhang C., Lei J., Xu H., Leng Z., Li T. (2019). LncTarD: A manually-curated database of experimentally-supported functional lncRNA–target regulations in human diseases. Nucleic Acids Res..

[225] Ma L., Cao J., Liu L., Du Q., Li Z., Zou D., Bajic V.B., Zhang Z. (2019). LncBook: A curated knowledgebase of human long non-coding RNAs. Nucleic Acids Res..

[226] Huang Z., Shi J., Gao Y., Cui C., Zhang S., Li J., Zhou Y., Cui Q. (2019). HMDD v3.0: A database for experimentally supported human microRNA–disease associations. Nucleic Acids Res..

[227] Yue M., Zhou D., Zhi H., Wang P., Zhang Y., Gao Y., Guo M., Li X., Wang Y., Zhang Y. (2018). MSDD: A manually curated database of experimentally supported associations among miRNAs, SNPs and human diseases. Nucleic Acids Res..

[228] Das S.S., Saha P., Chakravorty N. (2018). miRwayDB: A database for experimentally validated microRNA-pathway associations in pathophysiological conditions. Database.

[229] Jakhar D., Kaur I. (2020). Artificial intelligence, machine learning and deep learning: Definitions and differences. Clin. Exp. Dermatol..

[230] Luxton D.D. (2016). An Introduction to Artificial Intelligence in Behavioral and Mental Health Care.

[231] Handelman G.S., Kok H.K., Chandra R.V., Razavi A.H., Lee M.J., Asadi H. (2018). eDoctor: Machine learning and the future of medicine. J. Intern. Med..

[232] Obermeyer Z., Emanuel E.J. (2016). Predicting the Future—Big Data, Machine Learning, and Clinical Medicine. N. Engl. J. Med..

[233] Bi Q., Goodman K.E., Kaminsky J., Lessler J. (2019). What is Machine Learning? A Primer for the Epidemiologist. Am. J. Epidemiol..

[234] Senders J.T., Staples P.C., Karhade A.V., Zaki M.M., Gormley W.B., Broekman M.L., Smith T.R., Arnaout O. (2018). Machine Learning and Neurosurgical Outcome Prediction: A Systematic Review. World Neurosurg..

[235] Carpenter K. (2018). Machine Learning-based Virtual Screening and Its Applications to Alzheimer’s Drug Discovery: A Review. Curr. Pharm. Des..

[236] Stewart J., Sprivulis P., Dwivedi G. (2018). Artificial intelligence and machine learning in emergency medicine. Emerg. Med. Australas..

[237] Álvarez J.D., Matias-Guiu J.A., Cabrera-Martín M.N., Risco-Martín J.L., Ayala J.L. (2019). An application of machine learning with feature selection to improve diagnosis and classification of neurodegenerative disorders. BMC Bioinform..

[238] Shew M., New J., Wichova H., Koestler D.C., Staecker H. (2019). Using Machine Learning to Predict Sensorineural Hearing Loss Based on Perilymph Micro RNA Expression Profile. Sci. Rep..

[239] Rashidi H.H., Tran N.K., Betts E.V., Howell L.P., Green R. (2019). Artificial Intelligence and Machine Learning in Pathology: The Present Landscape of Supervised Methods. Acad. Pathol..

[240] DeGregory K.W., Kuiper P., DeSilvio T., Pleuss J.D., Miller R., Roginski J.W., Fisher C.B., Harness D., Viswanath S., Heymsfield S.B. (2018). A review of machine learning in obesity. Obes. Rev..

[241] Sidey-Gibbons J.A.M., Sidey-Gibbons C.J. (2019). Machine learning in medicine: A practical introduction. BMC Med. Res. Methodol..

[242] Paquin F., Rivnay J., Salleo A., Stingelin N., Silva C. (2015). Multi-phase semicrystalline microstructures drive exciton dissociation in neat plastic semiconductors. J. Mater. Chem. C.

[243] Vaidya J., Kantarcıoğlu M., Clifton C. (2008). Privacy-preserving Naïve Bayes classification. VLDB J..

[244] Renganathan V. (2015). Overview of artifi cial neural network models in the biomedical domain. Bratisl. Med. J..

[245] Kriegeskorte N., Golan T. (2019). Neural network models and deep learning. Curr. Biol..

[246] Kodinariya T.M., Makwana P.R. (2013). Review on determining number of Cluster in K-Means Clustering. Int. J. Adv. Res. Comput. Sci. Manag. Stud..

[247] Lin Y., Zhu X., Zheng Z., Dou Z., Zhou R. (2019). The individual identification method of wireless device based on dimensionality reduction and machine learning. J. Supercomput..

[248] (2013). Computational Toxicology. Methods Mol. Biol..

[249] Smith L.I. (2002). A Tutorial on Principal Components Analysis (Computer Science Technical Report No. OUCS-2002-12). http://hdl.handle.net/10523/7534.

[250] Dos Santos M.C.T., Barreto-Sanz M.A., Correia B.R.S., Bell R., Widnall C., Perez L.T., Berteau C., Schulte C., Scheller D., Berg D. (2018). miRNA-based signatures in cerebrospinal fluid as potential diagnostic tools for early stage Parkinson’s disease. Oncotarget.

[251] Zhang Z. (2015). Long non-coding RNAs in Alzheimer’s disease. Curr. Top. Med. Chem..

[252] Leidinger P., Backes C., Deutscher S., Schmitt K., Mueller S.C., Frese K., Haas J., Ruprecht K., Paul F., Stähler C. (2013). A blood based 12-miRNA signature of Alzheimer disease patients. Genome Biol..

